# Supplementary material for: Structure of the 70S Ribosome from the Human Pathogen Acinetobacter baumannii in Complex with Clinically Relevant Antibiotics
Source: Structure. 2020 Oct 6;28(10):1087–1100.e3. doi: 10.1016/j.str.2020.08.004 (PMC7546915; doi:10.1016/j.str.2020.08.004)
Supplement: Document S1. Figures S1–S7 and Tables S1–S3 [file mmc1.pdf]

Structure, Volume *28*

Supplemental Information

Structure of the 70S Ribosome from the Human Pathogen *Acinetobacter baumannii* in Complex with Clinically Relevant Antibiotics

David Nicholson, Thomas A. Edwards, Alex J. O'Neill, and Neil A. Ranson

Table S1. **CryoEM data collection statistics, related to Figures 1-5.**

	**amikacin-ribosome**	**tigecycline-ribosome**
microscope	Titan Krios	Titan Krios
camera	K2 Summit	Falcon 3EC
collection mode	counting	linear
voltage (kV)	300	300
nominal magnification	130,000	75,000
pixel size (Å/px)	1.07	1.065
defocus range (µm)	(-0.8)–(-2.7)	(-0.8)–(-2.6)
exposure time (s)	10	1.1
total dose ($e^-/Å^2$)	58	61.78
dose per frame ($e^-/Å^2$)	1.16	1.44
micrographs collected	2717	6228
micrographs with good ice	554	6228
number of particles	51958	231159
symmetry imposed	C1	C1
final resolution (Å)	2.8	2.6

Table S2. **Atomic model details. Related to Figure 1.**

rRNA chains					
**chain ID**	**rRNA name**	**GenBank accession number**	**locus tag**	**modelled residues**	**corresponding EM map**
1	23S	KL810966	DJ41_1033	2-868; 891-1034; 1101-1511; 1535-1712; 1726-2086; 2181-2891	50S
2	16S 5’	KL810966	DJ41_1036	3-77; 90-199; 210-837; 843-923	30S body
3	16S central	KL810966	DJ41_1036	924-1022; 1031-1384	30S head
4	16S 3’	KL810966	DJ41_1036	1385-1530	30S body
5	5S	KL810966	DJ41_1032	2-116	50S

protein chains						
**chain ID**	**protein name**	**UniProt accession number**	**full size (residues)**	**modelled residues**	**corresponding EM map**	**notes**
A	uL2	D0CD00	274	2-273	50S	
B	uL3	D0CCZ7	212	2-212	50S	
C	uL4	D0CCZ8	200	2-200 (2-12 no side chains)	50S	
D	uL5	D0CD09	178	(2-177 no side chains)	50S	
E	uL6	D0CD12	177	2-176	50S	
F	uL13	D0CG35	142	1-142	50S	
G	uL14	D0CD07	122	1-122	50S	
H	uL15	D0CD16	146	2-145	50S	
I	uL16	D0CD04	137	1-137	50S	
J	bL17	D0CD23	125	1-119	50S	
K	uL18	D0CD13	116	2-116	50S	
L	bL19	D0CCR8	122	2-118	50S	
M	bL20	D0CA76	119	2-118	50S	
N	bL21	D0CDQ6	103	1-103	50S	
O	uL22	D0CD02	110	2-110	50S	
P	uL23	D0CCZ9	106	1-92	50S	
Q	uL24	D0CD08	105	2-103	50S	
R	bL25	D0C9L7	98	3-98	50S	
S	bL27	D0CDQ7	85	9-84	50S	
T	bL28	D0CAL0	78	2-78	50S	
U	uL29	D0CD05	65	2-62	50S	

V	uL30	D0CD15	58	1-57	50S	
W	bL31	D0CBZ8	74	(1-43 no side chains)	50S	tigecycline model only
X	bL32	D0C9K5	61	2-55	50S	
Y	bL33	D0CAL1	51	1-51	50S	
Z	bL34	D0CG06	44	1-44	50S	
a	bL35	D0CA77	64	2-64	50S	
b	bL36	D0CD18	38	1-38	50S	
c	uS2	D0CC74	250	(8-226 no side chains)	30S body	
d	uS3	D0CD03	250	2-211	30S head	
e	uS4	D0CD21	208	2-208 (23-30; 43-51 no side chains)	30S body	
f	uS5	D0CD14	165	10-164	30S body	
g	bS6	D0C5Z0	127	1-103	30S body	
h	uS7	D0C9P7	156	3-70; 97-145 (126-145 no side chains)	30S head	
i	uS8	D0CD11	131	2-131	30S body	
j	uS9	D0CG36	128	2-128	30S head	
k	uS10	D0CCZ6	103	4-103	30S head	
l	uS11	D0CD20	128	15-128	30S body	
m	uS12	D0C9P6	124	2-123	30S body	
n	uS13	D0CD19	118	2-116	30S head	
o	uS14	D0CD10	101	2-101	30S head	
p	uS15	D0CAU9	89	2-89	30S body	
q	bS16	D0CCR5	83	1-80	30S body	
r	uS17	D0CD06	85	5-83	30S body	
s	bS18	D0C5Y9	75	21-73	30S body	
t	uS19	D0CD01	91	2-84	30S head	
u	bS20	D0C7N1	88	2-87	30S body	
v	bS21	D0C5Q3	71	2-61 (2-36 no side chains)	30S body	

other chains			
**chain ID**	**name**	**corresponding EM map**	**notes**
6	tRNA 5’	50S	residues 2-6 from PDB 5AFI E-site fMet-tRNA, probably actually a mixture of tRNAs
7	tRNA central	30S head	residues 26-45 from PDB 5AFI E-site fMet-tRNA, probably actually a mixture of tRNAs
8	tRNA 3’	50S	residues 69-76 from PDB 5AFI E-site fMet-tRNA, probably actually a mixture of tRNAs
9	mRNA	30S head	4 nucleotide polyU mRNA model, probably actually a mixture of mRNAs

unmodelled proteins
uL1
bL9
uL10
uL11
bL12
bS1

Table S3. **Unique structural features of the *A. baumannii* ribosome. Related to Figure 2.**

chain	*A. baumannii* residue number	*E. coli* residue number	rRNA helix	details	scale of difference	quality of density
**rRNAs**						
23S	60-64	60-64	H6	different fold	minor	good
	99-105	99-105	H7	different fold	major	good
	135-147	135-147	H9	different fold	minor	fair
	263-284	263-289	H18	deletion	major	fair
	349-365	354-373	H18	deletion	major	good
	634-639	643-648	H31	different fold	minor	good
	921-925	930-935	H38	deletion	minor	good
	1130-1134	1140-1143	H41-42	insertion	minor	good
	1490-1503	1502-1514	H58	insertion	minor	fair
	1701-1738	1710-1749	H63	deletion	major	positions 1713-1725 not resolved
16S	70-98	68-102	h6	deletion	major	positions 78-89 not resolved
	153-165	157-169	h8	different fold	minor	fair
	175-189	179-193	h9	different fold	major	good
	447-479	451-482	h17	insertion	minor	fair
	1065-1081	1068-1084	h35-36	different fold	minor	good
	1252-1256	1255-1259	h41	different fold	minor	good
	1436-1440	1439-1443	h44	different fold	minor	fair
5S	5'- and 3'-ends	5'- and 3'-ends		shorter	minor	fair
	82-91	83-93	helix IV	deletion	major	good
**proteins**						
uL3	85-96	84-93		insertion	major	good
	104-109	101-106		different fold	major	good
uL6	15-23	15-23		different fold	minor	fair
	54-60	54-60		different fold	major	fair
uL14	88-92	88-93		deletion	minor	good
uL16	20-24	20-23		insertion	minor	good
uL18	54-64	53-65		deletion	major	fair
bL19	N-terminus	N-terminus		extension	major	good
uL22	C-terminus	C-terminus		deletion	minor	good
uL23	N-terminus	N-terminus		deletion	minor	fair
	65-74	66-75		different fold	minor	good
uL24	N-terminus	N-terminus		deletion	minor	good
	27-31	28-33		deletion	minor	good
	C-terminus	C-terminus		extension	minor	fair
bL25	11-16	9-12		insertion	minor	good
	83-90	79-86		different fold	minor	good
bL32	23-33	23-34		deletion	major	good
	51-55	52-56		different fold	minor	good
bL33	N-terminus	N-terminus		deletion	major	good
bL34	C-terminus	C-terminus		deletion	major	good
bL35	17-21	17-22		deletion	minor	good
uS3	59-65	59-65		different fold	minor	good
uS4	31-37	31-36		insertion	major	fair
	82-88	80-86		different fold	minor	good

uS8	53-57	53-56		insertion	minor	good
uS15	N-terminus	N-terminus		different fold	minor	good
bS16	44-50	44-50		different fold	minor	good
uS17	N-terminus	N-terminus		different fold	minor	good

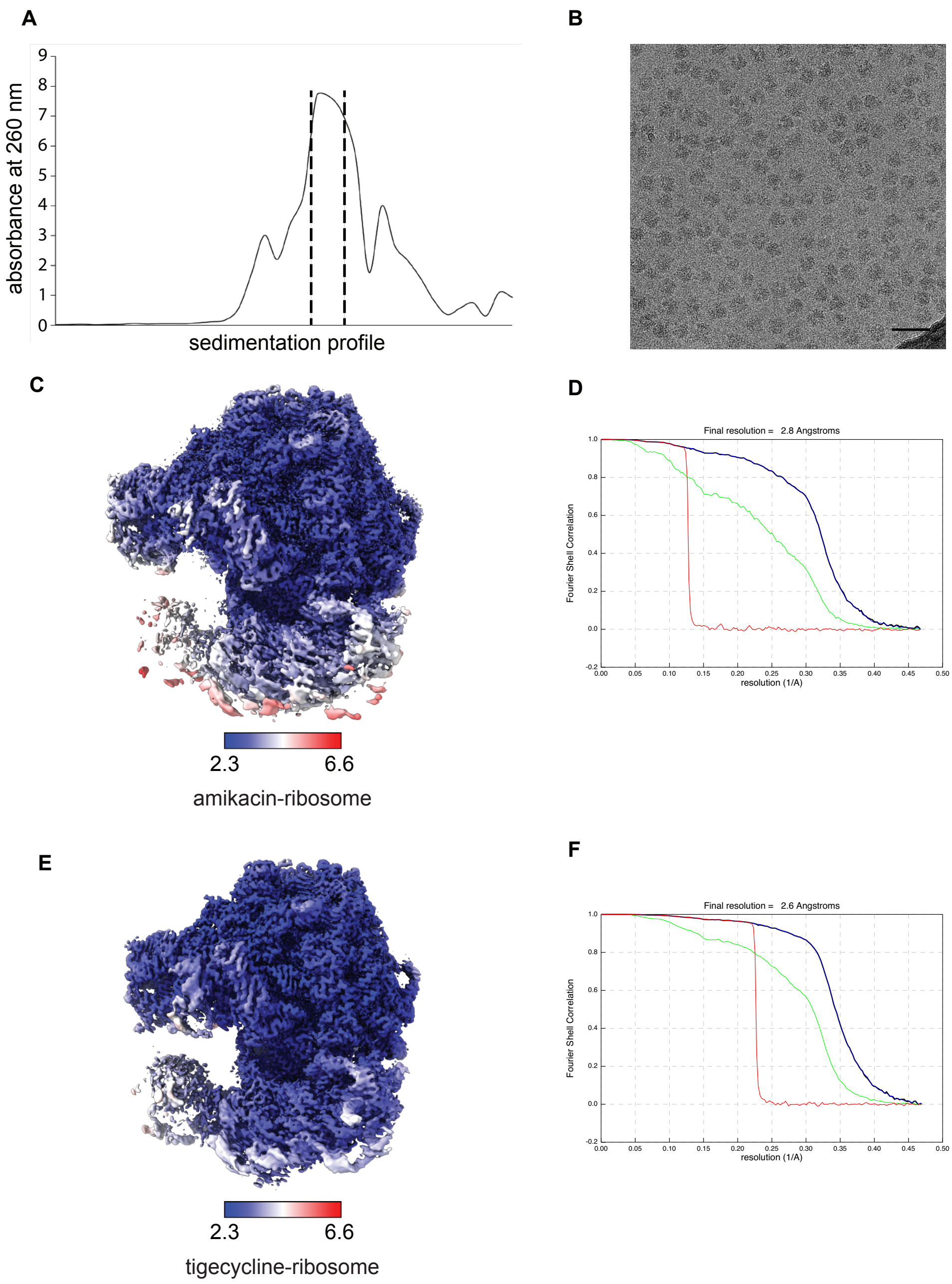

Figure S1. **Sample characterisation and cryoEM image processing, related to Figures 1-5.** A) Sucrose gradient A_{260} sedimentation profile, showing the peak where fractions were collected. B) Representative cryoEM micrograph of the *A. baumannii* ribosome-amikacin sample, scale bar 50 nm. C) CryoEM reconstruction of the amikacin-ribosome structure, filtered by local resolution. D) FSC curves as a function of resolution for the amikacin-ribosome struc-ture. The resolution that corresponds to an FSC coefficient of 0.143 is 2.8 Å. E) CryoEM reconstruction of the *A. baumannii* ribosome-tigecycline structure, filtered by local resolution. F) FSC curves as a function of resolution for the tigecycline-ribosome structure. The resolution that corresponds to an FSC coefficient of 0.143 is 2.6 Å. Maps coloured by local resolution, from 2.3 Å (blue) to 6.6 Å (red). FSC curves are shown for phase-randomised maps (red), unmasked maps (green), masked maps (blue), and masked maps after correction for mask convolution effects (black).

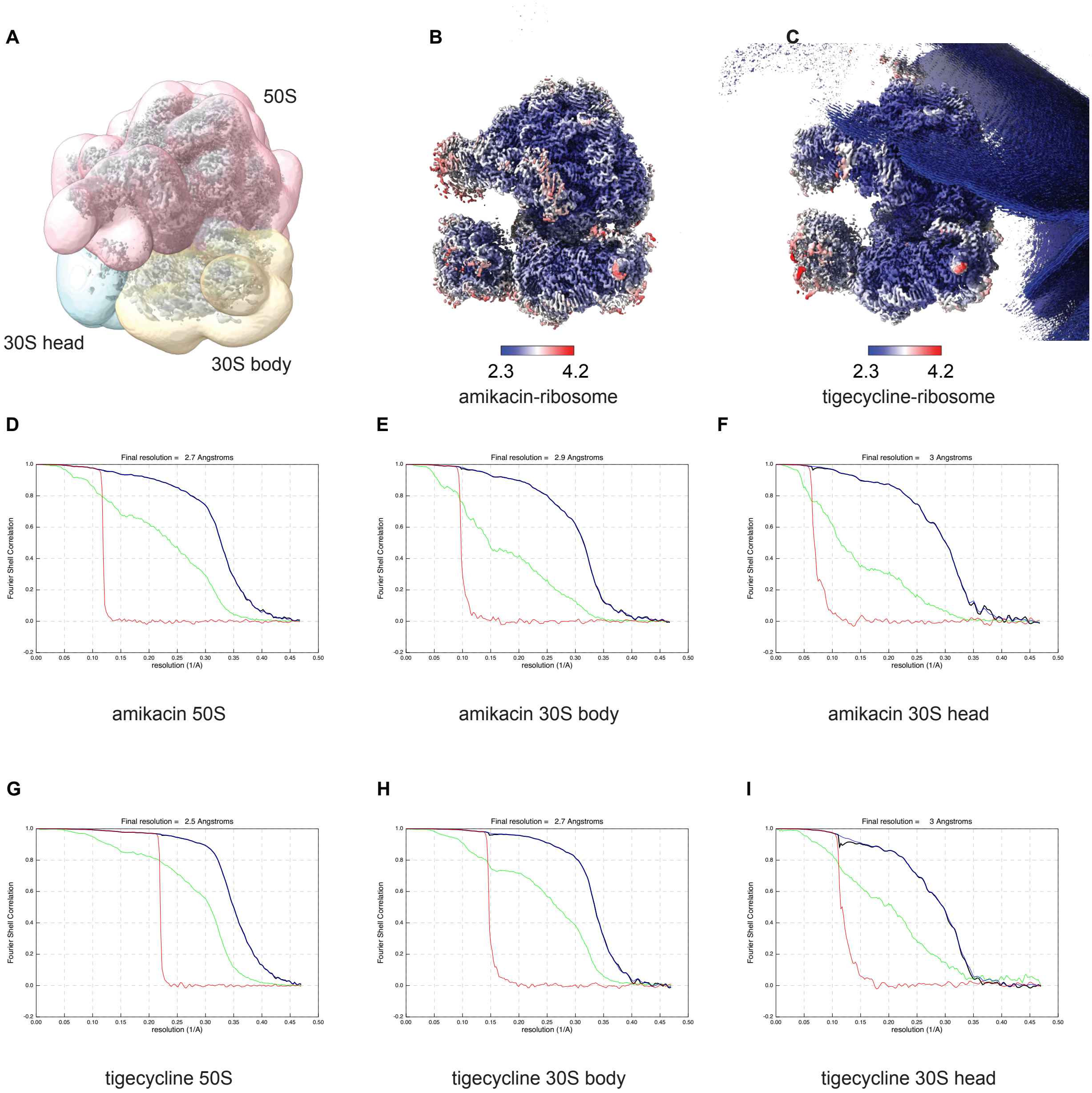

Figure S2. **CryoEM multibody refinement, related to Figure 1-5.** A) Masks used to specify the 50S (pink), 30S body (brown) and 30S head (blue) rigid bodies, shown around the pre-multibody consensus reconstruction of the *A. baumannii* ribosome-amikacin complex (grey). B) CryoEM reconstructions of the three bodies comprising the amikacin-ri-bosome structure after multibody refinement, filtered by local resolution. C) CryoEM reconstructions of the three bodies comprising the tigecycline-ribosome structure after multibody refinement, filtered by local resolution. Maps coloured by local resolution, from 2.3 Å (blue) to 4.2 Å (red). Note that padding in Fourier space was not performed in order to save memory, resulting in artefacts around the edge of the box. This noise was masked out before undertaking model building and refinement. D-F) FSC curves as a function of resolution for the amika-cin-ribosome multibody structures. G-I) FSC curves as a function of resolution for the tigecycline-ribosome multi-body structures. FSC curves are shown for phase-randomised maps (red), unmasked maps (green), masked maps (blue), and masked maps after correction for mask convolution effects (black).

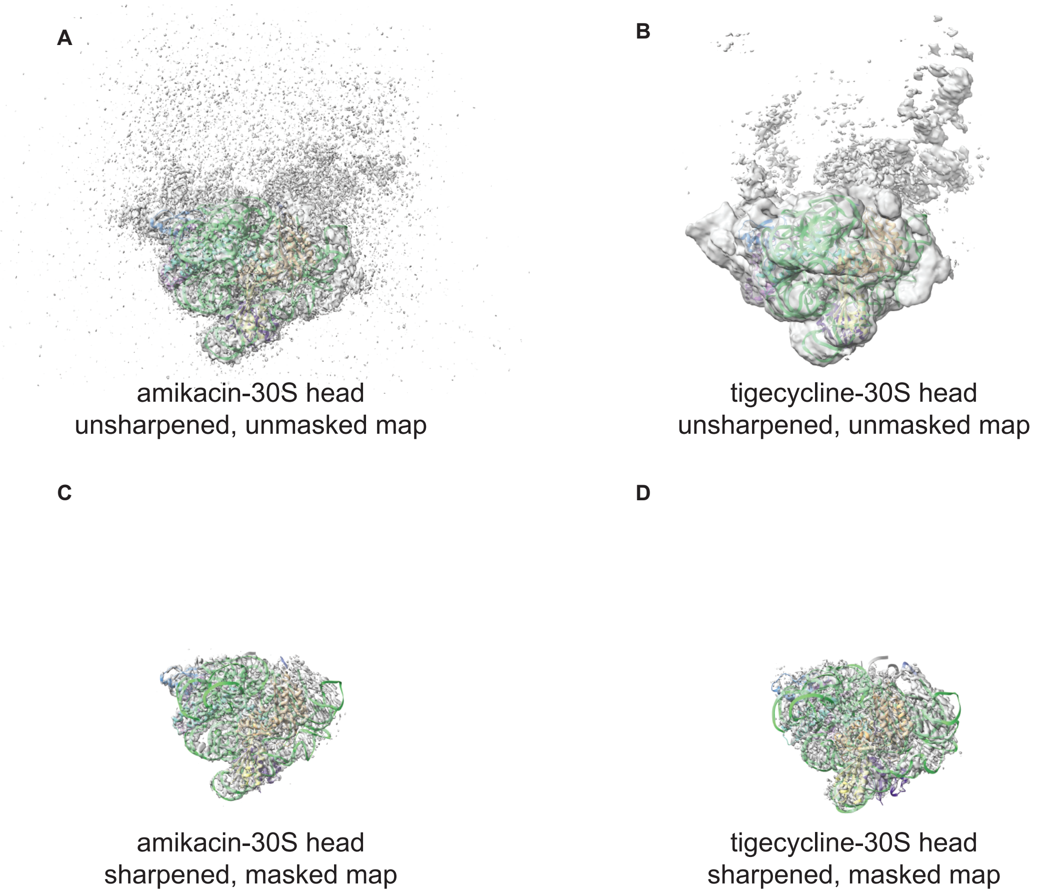

Figure S3. **Comparison of the 30S head EM density of the *A. baumannii* ribosome-amikacin and *A. baumannii* ribosome-tigecycline structures, related to Figure 1-5.** A) Atomic model and EM density of the 30S head of the amika-cin-ribosome complex. B) Atomic model and EM density of the 30S head of the tigecycline-ribosome complex. Semi-transparent grey densities correspond to the unsharpened and unmasked outputs of multibody refinement. C) Atomic model and EM of the 30S head of the amikacin-ribosome complex. D) Atomic model and EM density of the 30S head of the tigecycline-ribosome complex. Semi-transparent grey densities correspond to the sharp-ened and masked maps after post-processing of the multibody refinement outputs.

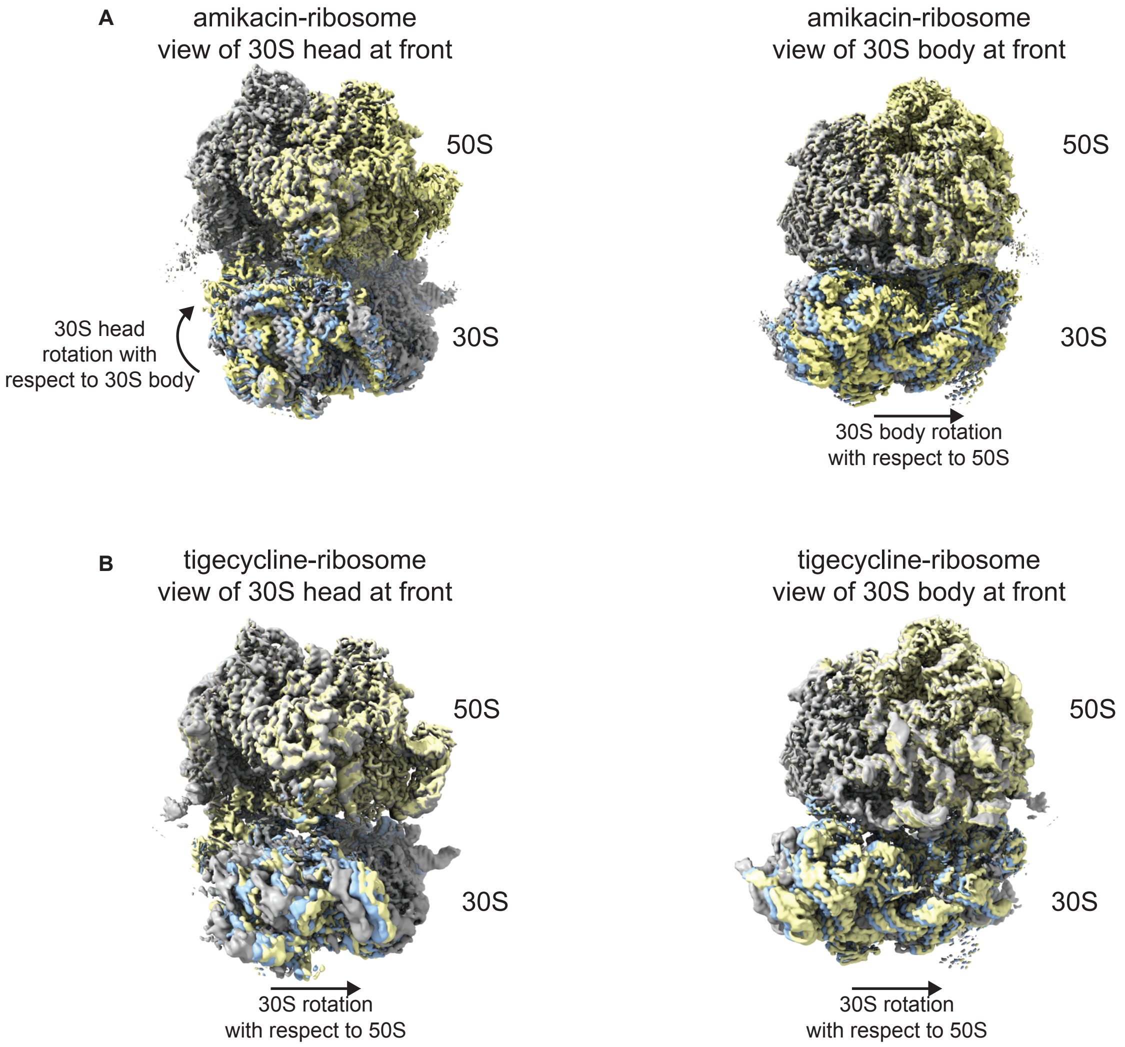

Figure S4. **Comparison of the components contributing the largest variations to the data for the *A. baumannii* ribosome-amikacin and ribosome-tigecycline complexes, related to Figure 1-5.** A) The principle component describ-ing 27% of the variation in the data for the amikacin-ribosome complex. Movement is described along a trajecto-ry from grey to blue to yellow EM density. This component describes a rotation of the 30S head as well as inter-subunit rotation between the 50S and 30S body. B) The principle component describing 28% of the varia-tion in the data for the tigecycline-ribosome complex. Movement is described along a trajectory from grey to blue to yellow EM density. This component describes predominantly inter-subunit rotation between the 50S and 30S.

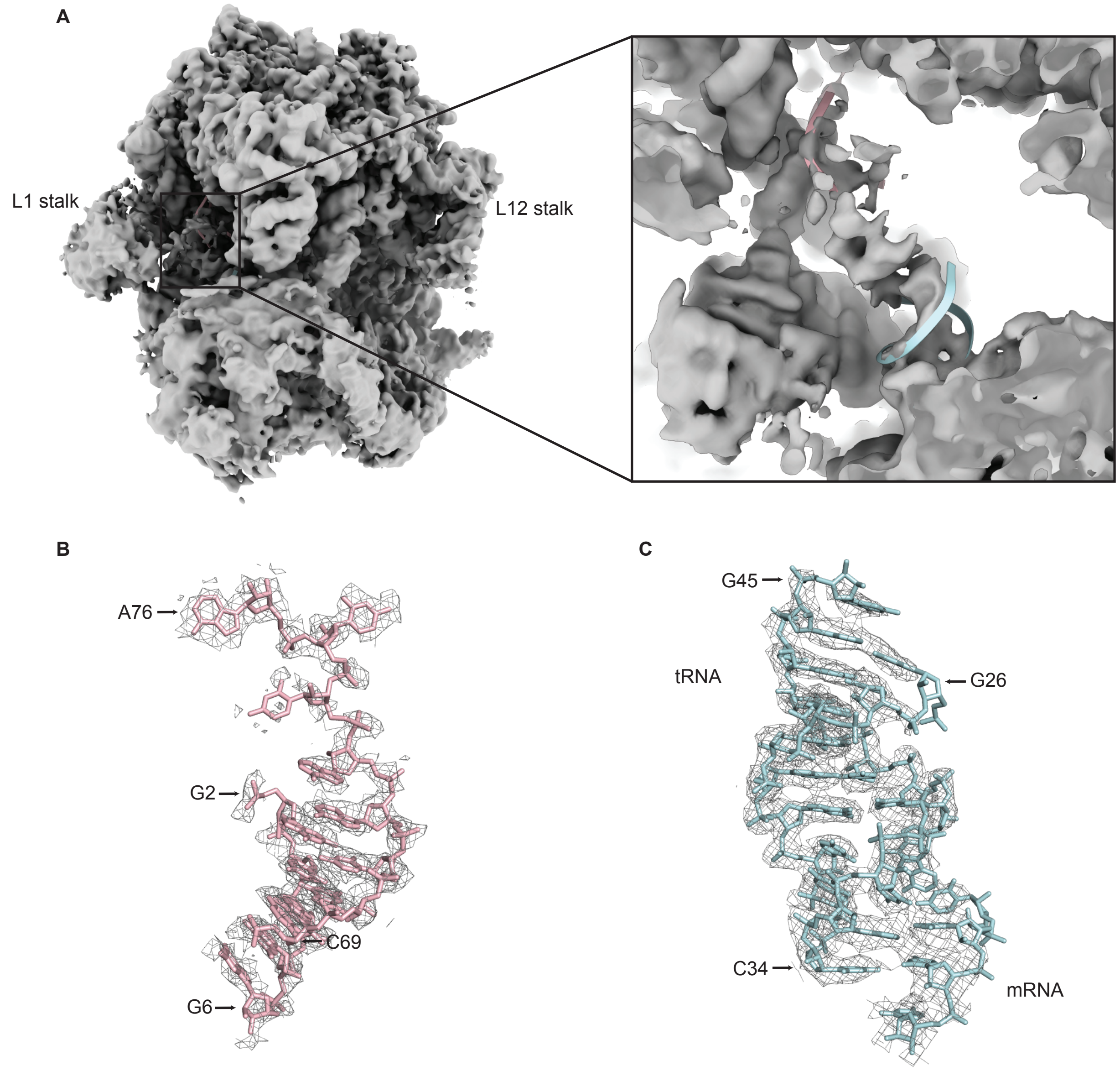

Figure S5. **CryoEM density and atomic model of E-site tRNA and mRNA, related to Figures 1-3.** A) Unsharpened consensus recon-struction of the amikacin-ribosome complex lowpass filtered to 5 Å. Weak cryoEM density is seen in the E-site of the ribosome that can fit tRNA and mRNA (ribbon). B) Atomic model of the 5' and 3' ends of E-site tRNA (light pink), derived from an E. coli fMet-tRNA starting model and fitted and refined into the sharpened and masked 50S multibody reconstruction (grey mesh). C) Atomic model of the anticodon stem-loop of the E-site tRNA, derived from an E. coli fMet-tRNA starting model, and a short polyuridine mRNA (light blue), fitted and refined into the sharpened and masked 30S head multibody reconstruction (grey mesh).

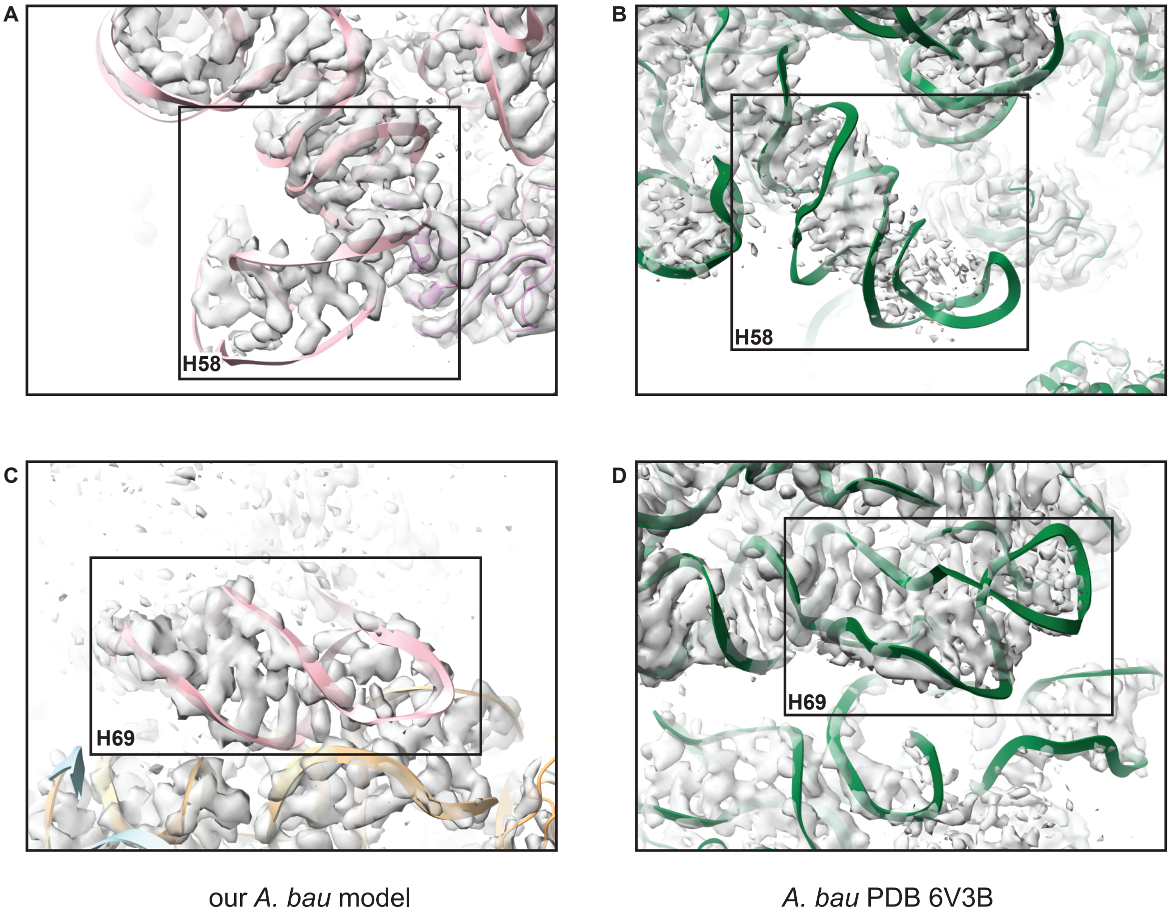

Figure S6. **EM density of 23S rRNA H58 and H69 of the *A. baumannii* ATCC 19606 and *A. baumannii* AB0057 ribosomes. Related to Figure 4.** A) H58 of the *A. baumannii* amikacin-ribosome model presented in this paper (pink, strain ATCC 19606), shown in the consensus EM map filtered by local resolution (grey). B) H58 of the alternative *A. baumannii* ribosome model (green, strain AB0057, PDB 6V3B) shown in the corresponding EM map (grey, EMD-21032). C) H69 of the *A. baumannii* amikacin-ribosome model presented in this paper (pink, strain ATCC 19606), shown in the consensus EM map filtered by local resolution (grey). D) H69 of the alternative *A. baumannii* ribosome model (green, strain AB0057, PDB 6V3B) shown in the corresponding EM map (grey, EMD-21032). In all cases, the EM density shows that the different helix conformations shown in Figure 4 correspond to the data and are not modelling errors.

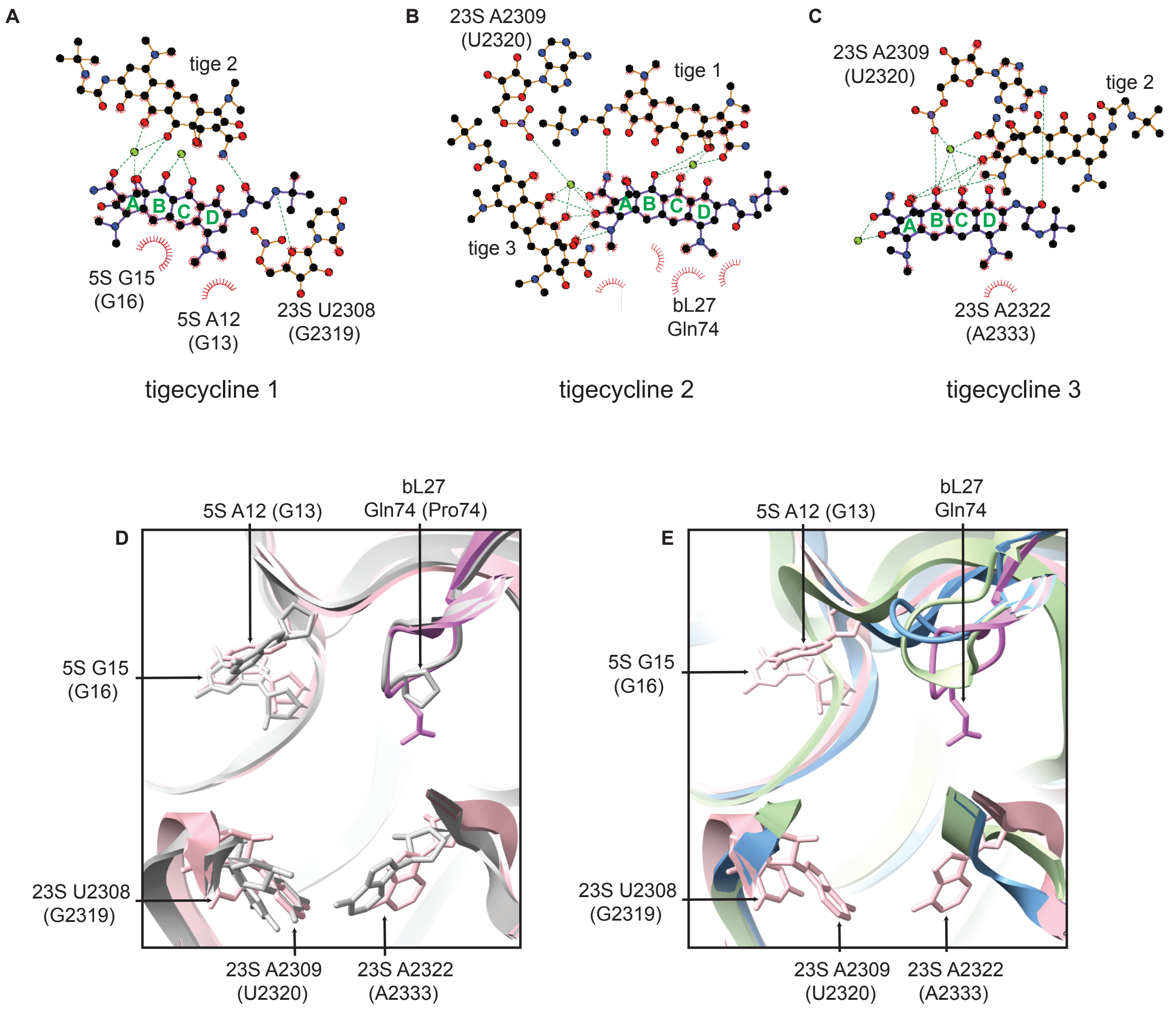

Figure S7. **The secondary tigecycline binding site. Related to Figure 7.** A-C) 2D diagrams representing the interactions that the 3 tigecycline molecules make with the surrounding ribosome. Red spikes represent hydrophobic interactions and green dashes represent polar or ionic interactions. LigPlot+ (Laskowski and Swindells, 2011) was used to generate the diagrams, which were then adapted to highlight the major interactions calculated by Arpeggio (Jubb et al., 2017). D) Atomic model of the *A. baumannii* ribosome-amikacin complex showing the secondary tigecycline binding site (with no drug bound), with residues that interact with tigecycline highlighted (pink), and atomic model of the *E. coli* ribosome with the equivalent *E. coli* residues highlighted (grey, PDB 5MDZ). Many of the residues proposed to be involved in tigecycline binding take similar conformations in the two structures. E) Atomic models of the secondary tigecycline binding site in the *A. baumannii* ribosome-amikacin complex (pink), *S. aureus* ribosome (blue, PDB 5LI0) and *T. thermophilus* ribosome (green, PDB 5E81). The rRNA surrounding the binding site and the loop in bL27, proposed to be involved in tigecycline binding, take up quite different folds in these structures. *E. coli* numbering is shown in parentheses.
