# Supplementary material for: Structure of the 70S Ribosome from the Human Pathogen Acinetobacter baumannii in Complex with Clinically Relevant Antibiotics
Source: Structure. 2020 Oct 6;28(10):1087–1100.e3. doi: 10.1016/j.str.2020.08.004 (PMC7546915; doi:10.1016/j.str.2020.08.004)
Supplement: Document S2. Article plus Supplemental Information [file mmc2.pdf]

Structure

Article

Structure of the 70S Ribosome from the Human Pathogen *Acinetobacter baumannii* in Complex with Clinically Relevant Antibiotics

Graphical Abstract

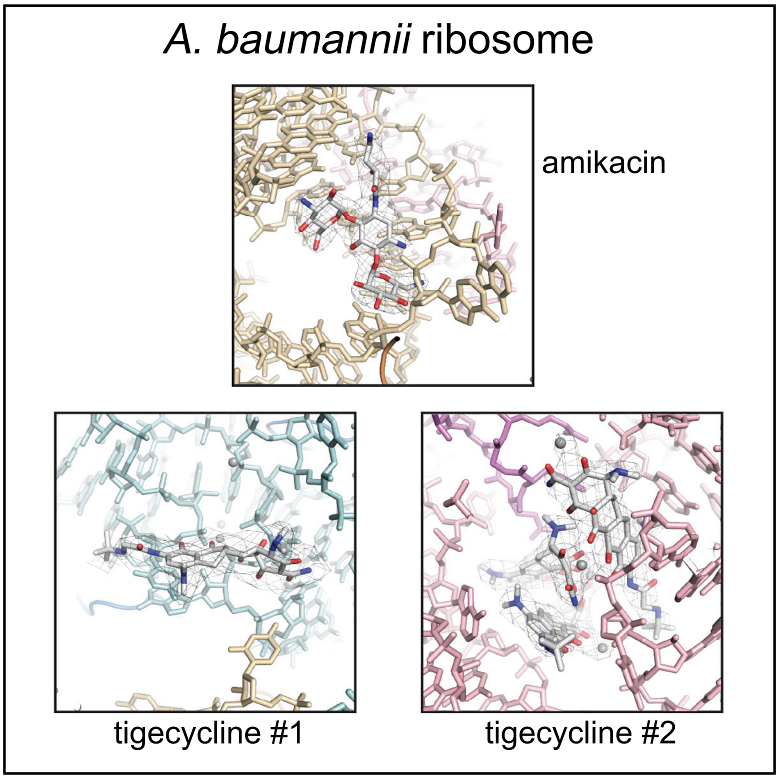

Highlights

- Cryo-EM structures of the ribosome from the pathogenic bacteria *A. baumannii*
- Unique structural features compared with other bacterial ribosomes
- The site and mode of binding of amikacin and tigecycline to this ribosome
- A putative alternative tigecycline-binding site at the 50S central protuberance

Authors

David Nicholson, Thomas A. Edwards, Alex J. O'Neill, Neil A. Ranson

Correspondence

t.a.edwards@leeds.ac.uk (T.A.E.),
a.j.oneill@leeds.ac.uk (A.J.O.),
n.a.ranson@leeds.ac.uk (N.A.R.)

In Brief

Nicholson et al. present high-resolution cryo-EM structures of the ribosome from the pathogenic bacteria *Acinetobacter baumannii* in complex with the clinically relevant antibiotics amikacin and tigecycline, revealing species-specific features and modes of drug interactions. These structures pave the way for the design of new inhibitors to address multidrug-resistant *A. baumannii* infections.

Nicholson et al., 2020, Structure *28*, 1087–1100
October 6, 2020 © 2020 The Authors. Published by Elsevier Ltd.
https://doi.org/10.1016/j.str.2020.08.004

CellPress

Structure

CellPress
OPEN ACCESS

Article

Structure of the 70S Ribosome from the Human Pathogen *Acinetobacter baumannii* in Complex with Clinically Relevant Antibiotics

David Nicholson,[1] Thomas A. Edwards,[1,*] Alex J. O'Neill,[1,*] and Neil A. Ranson[1,2,*]
[1]Astbury Centre for Structural Molecular Biology, School of Molecular & Cellular Biology, Faculty of Biological Sciences, University of Leeds, Leeds LS2 9JT, UK
[2]Lead Contact
*Correspondence: t.a.edwards@leeds.ac.uk (T.A.E.), a.j.oneill@leeds.ac.uk (A.J.O.), n.a.ranson@leeds.ac.uk (N.A.R.)
https://doi.org/10.1016/j.str.2020.08.004

SUMMARY

Acinetobacter baumannii is a Gram-negative bacterium primarily associated with hospital-acquired, often multidrug-resistant (MDR) infections. The ribosome-targeting antibiotics amikacin and tigecycline are among the limited arsenal of drugs available for treatment of such infections. We present high-resolution structures of the 70S ribosome from *A. baumannii* in complex with these antibiotics, as determined by cryoelectron microscopy. Comparison with the ribosomes of other bacteria reveals several unique structural features at functionally important sites, including around the exit of the polypeptide tunnel and the periphery of the subunit interface. The structures also reveal the mode and site of interaction of these drugs with the ribosome. This work paves the way for the design of new inhibitors of translation to address infections caused by MDR *A. baumannii*.

INTRODUCTION

Acinetobacter baumannii is a formidable opportunistic pathogen and an important cause of hospital-acquired infection. This bacterium predominantly affects immunocompromised patients who require prolonged hospital stays, in whom it can cause pneumonia, meningitis, and infections of the blood, urinary tract, and skin and soft tissue (Dijkshoorn et al., 2007; Howard et al., 2012; Montefour et al., 2008; Peleg et al., 2008). As a Gram-negative bacterium equipped with an outer membrane and possessing an array of efflux transporters, *A. baumannii* is intrinsically well defended against antibacterial drugs. However, it also readily acquires novel resistance determinants through horizontal gene transfer, which has led to the rapid emergence of multidrug-resistant strains. These include strains resistant to most or all classes of antibiotics (Falagas and Bliziotis, 2007; Fournier et al., 2006; Perez et al., 2008; Potron et al., 2015). Accordingly, it has been classified as one of the ESKAPE pathogens, a designation reserved for those bacteria most commonly associated with multidrug resistance (Boucher et al., 2009).

The antibiotics available for treatment of *A. baumannii* infections in the clinic include beta-lactams, polymyxins, and ribosome-targeting antibiotics such as the aminoglycosides (e.g., amikacin) and tigecycline (a third-generation tetracycline derivative) (Fishbain and Peleg, 2010). However, the effectiveness of even these agents is diminishing, and there are few recently approved drugs or candidates in late-stage development to replace them (Asif et al., 2018; Baron et al., 2016; Perez et al., 2007; Poirel and Nordmann, 2006; Wright et al., 2017). Consequently, the World Health Organization has placed carbapenem-resistant *A. baumannii* in the top tier of their priority pathogens list for research and development of new antibiotics (Tacconelli et al., 2018). A more detailed molecular understanding of how current antibiotics bind their targets and exert their inhibitory effects on this pathogen may aid the design and development of such drugs.

The bacterial ribosome is one such antibiotic target (Wilson, 2014). Although structures of ribosomes and antibiotic-ribosome complexes from a range of bacterial species have been determined (Ban et al., 2000; Brodersen et al., 2000; Carter et al., 2000; Schluenzen et al., 2000; Schlunzen et al., 2001; Wimberly et al., 2000), only three species of pathogenic bacteria have known structures of drug-bound ribosomes, namely *E. coli* (Borovinskaya et al., 2007; Schuwirth et al., 2006), *Staphylococcus aureus* (Eyal et al., 2015; Halfon et al., 2019b), and *Mycobacterium tuberculosis* (Yang et al., 2017). Recently, the structures of the ribosomes from *Pseudomonas aeruginosa* (Halfon et al., 2019a) and *A. baumannii* (Morgan et al., 2020) were solved, but these did not include bound antibiotics. Expanding our repertoire of bacterial ribosome and antibiotic-ribosome complex structures will improve our understanding of species-specific translation and translation inhibition mechanisms, and inform the development of new antibiotics with improved activity against specific pathogens (Khusainov et al., 2016; Wilson, 2014). The variation in ribosomes between species can be substantial (e.g., in the form of rRNA expansion

This is an open access article under the CC BY license (http://creativecommons.org/licenses/by/4.0/).

segments [Yang et al., 2017], unique ribosomal proteins [Hentschel et al., 2017; Yang et al., 2017], and protein paralogs [Khusainov et al., 2016]). However, variation can also be more subtle (e.g., different protein and rRNA folds, insertions, and deletions [Eyal et al., 2015] and chemical modifications [Byrgazov et al., 2013; Polikanov et al., 2015]). Specific examples include differences in helix h26 of the 16S rRNA, a region that interacts with the Shine-Dalgarno sequence and that varies slightly in length between ribosomes from *Thermus thermophilus*, *E. coli*, *Bacillus subtilis*, and *S. aureus* (Khusainov et al., 2016), and protein uL22, which lines the polypeptide exit tunnel and contains a single residue difference between *B. subtilis* and *E. coli* that leads to ribosome stalling during the translation of the MifM leader peptide in *B. subtilis* specifically (Sohmen et al., 2015). Even variations between ribosomes from different strains of the same species can have a significant functional impact, as seen for a strain of *P. aeruginosa* with a mutation in ribosomal protein uL6 that results in aminoglycoside resistance and ribosome instability (Halfon et al., 2019a).

In this study we present cryoelectron microscopy (cryo-EM) structures of the ribosome from *A. baumannii* in complex with the clinically relevant antibiotics amikacin and tigecycline, solved to high resolutions of 2.8 and 2.6 Å, respectively, allowing comparison with previous apo structures of the *A. baumannii* ribosome (Morgan et al., 2020). Structural comparison with ribosomes from other bacteria identifies several unique structural features, including differences around the exit of the polypeptide tunnel and at the subunit interface. These structures also reveal the molecular detail of interactions of amikacin and tigecycline with the *A. baumannii* ribosome and suggest the existence of an alternative tigecycline-binding site within the 50S subunit. Collectively, these structures contribute toward a greater understanding of species-specific translation mechanisms and provide a platform for the design and development of novel antibiotics needed to treat increasingly drug-resistant *A. baumannii* infections.

RESULTS AND DISCUSSION

Structure Determination of the 70S Ribosome from *A. baumannii*

Ribosomes were extracted from the *A. baumannii* type strain ATCC 19606 and then purified by sucrose cushion and sucrose gradient centrifugation. Fractions corresponding to the dominant peak in the sucrose gradient sedimentation profile were collected (Figure S1A). The purified ribosomes were incubated with either amikacin or tigecycline, and visually inspected by cryo-EM to confirm the presence of intact 70S ribosomes (Figure S1B). Single-particle cryo-EM analysis was performed to reconstruct structures of the amikacin-ribosome and tigecycline-ribosome complexes to resolutions of 2.8 and 2.6 Å, respectively (Table S1 and Figure S1). The local resolution of the reconstructions ranges from ~2.3 Å in the core of the 50S subunit to >6 Å in the flexible peripheral regions of the ribosome. Image alignment was dominated by the larger 50S subunit, leaving the smaller 30S subunit, particularly its head, poorly resolved, due to movements in the ribosome necessary to facilitate the translocation of the tRNA-mRNA complex. These include intersubunit rotation between the 50S and the 30S subunits (Cornish et al., 2008) and 30S head swiveling (Ratje et al., 2010). Therefore, for each structure, the 50S subunit, the body of the 30S subunit, and the head of the 30S subunit were treated as three independent rigid bodies and their reconstructions refined to nominal resolutions of 2.7, 2.9, and 3.0 Å for the amikacin-ribosome complex and 2.5, 2.7, and 3.0 Å for the tigecycline-ribosome complex, respectively (Figure S2). This multibody refinement procedure vastly improved the density of the 30S head in the amikacin-ribosome structure, making it amenable to model building (Figures S1C and S2B), but was less successful for the tigecycline-ribosome structure (Figures S1E and S2C). Note that padding in Fourier space was not performed in order to save computer memory, resulting in artifacts around the edge of the box in Figure S2C. This noise was masked out before undertaking model building and refinement. The 30S head of the tigecycline-ribosome structure contained significantly more density outside the expected region compared with the 30S head of the amikacin-ribosome structure, suggesting that the particle subtraction and focused refinement procedures within multibody refinement were less effective (Figures S3A and S3B). Furthermore, despite similar Fourier shell correlation-derived resolution estimates for the 30S head in the two structures, the masked and sharpened 30S head map of the tigecycline-ribosome structure was visually poorer than the corresponding map from the amikacin-ribosome complex, with protein side-chain and RNA base density less consistently resolvable across the whole map (Figures S3C and S3D), no matter the sharpening B factor used. This comparatively small improvement in the tigecycline-ribosome reconstruction could not be rectified, despite trying a number of different masks to define the rigid-body boundaries. The reason for the larger improvement in the 30S head in the amikacin-ribosome reconstruction compared with the 30S head in the tigecycline-ribosome reconstruction is unclear. One possible reason is that tigecycline locks the 30S head to the 30S body in a way similar to that of spectinomycin (Mohan et al., 2014), reducing 30S head rotation, and thus the improvement through multibody refinement would be expected to be less significant than if the 30S head were rotating more freely. Indeed, the 30S head density is more complete in the tigecycline-ribosome pre-multibody refinement reconstruction than in the corresponding amikacin-ribosome reconstruction (Figure S1), a phenomenon that could be explained by reduced 30S head swivel when tigecycline is bound. Furthermore, principal component analysis reveals that the largest variation in the data for the tigecycline-bound ribosome is due to 50S-30S intersubunit rotation, compared with a combination of 30S head rotation and intersubunit rotation for the amikacin-bound ribosome (Figure S4) Tigecycline-induced locking of the 30S head is plausible, considering that the primary tigecycline binding site is at the interface of the 30S head and body (Jenner et al., 2013).

Homology models based on experimental structures of *E. coli* ribosomal proteins and rRNA were fitted and refined into the *A. baumannii* antibiotic-ribosome cryo-EM multibody reconstructions (Table 1). The overall structure comprises a large 50S subunit, composed of 23S rRNA, 5S rRNA, and 28 ribosomal proteins, and a small 30S subunit composed of 16S rRNA and 20

CellPress
OPEN ACCESS

Table 1. Model Refinement and Validation Statistics[a]

	Amikacin-Ribosome 50S	Amikacin-Ribosome 30S Body	Amikacin-Ribosome 30S Head	Tigecycline-Ribosome 50S	Tigecycline-Ribosome 30S Body	Tigecycline-Ribosome 30S Head
Map resolution (Å) (FSC = 0.143)	2.7	2.9	3.0	2.5	2.7	3.0
Map sharpening B factor (Å^2)	−47.3	−55.7	−51.6	−82.5	−99.3	−131.4
Model Composition						
Non-hydrogen atoms	83,514	33,322	16,864	83,855	33,279	16,906
Protein residues	3,069	1,496	852	3,112	1,496	852
Nucleic acid residues	2,800	1,040	477	2,800	1,040	477
Metal ions	161	59	24	163	56	24
Ligand	none	amikacin	none	tigecycline ×3	none	tigecycline
General Validation						
CC[b] (model to map fit)	0.86	0.83	0.86	0.86	0.84	0.70
Clashscore	4.84	7.09	5.25	5.86	9.42	15.11
Root-mean-square Deviations						
Bond lengths (Å)	0.007	0.012	0.007	0.008	0.010	0.007
Bond angles (°)	0.775	0.974	0.702	0.730	0.794	0.853
Protein Geometry Validation						
Rotamer outliers (%)	7.08	11.02	7.15	5.70	8.22	11.24
Ramachandran outliers (%)	0.03	0.20	0.00	0.07	0.00	0.24
Ramachandran favored (%)	94.46	90.54	92.58	95.42	91.90	91.03
RNA Geometry Validation[c]						
Sugar pucker outliers (%)	0.64	0.38	0.21	0.57	0.38	0.42
Backbone conformation outliers (%)	17.14	20.48	17.19	15.89	19.71	22.85

[a]Obtained from Phenix refinement log unless otherwise stated.
[b]CC, correlation coefficient.
[c]Obtained from MolProbity Web server.

ribosomal proteins. These constituent parts form the recognizable structural elements of the ribosome, including the central protuberance, L1 stalk, and L12 stalk of the 50S subunit, and the head, body, platform, shoulder, and spur of the 30S subunit (Figure 1). At this resolution, most rRNA nucleobases (Figures 1A and 1D) and protein side chains (Figures 1B and 1C) can be distinguished in both subunits. Most rRNA residues were modeled, with missing regions mostly belonging to the flexible stalks of the 50S subunit, which had poorly resolved electron microscopy (EM) density, as has been seen previously in other ribosome structures. Of the 54 known core ribosomal proteins, 41 were modeled with side chains included, 7 had ambiguous or poor side-chain density and were modeled, at least in part, without side chains, and 6 were not modeled (Table S2). These unmodeled proteins are known to be located in flexible parts of the ribosome (uL1, bL9, uL10, uL11, and bL12), or to be loosely associated (bS1), and all had weak or non-existent density. Density corresponding to protein bL31, which bridges the two subunits, was resolved only in the tigecycline-ribosome reconstruction. Weak density was seen in the E site for mRNA (modeled as polyuridine) and tRNA (modeled as "E-site tRNA," derived from the E-site fMet-tRNA of PDB: 5AFI) (Figure S5). These densities likely correspond to a mixture of different tRNAs and mRNAs that remain associated with a subpopulation of ribosomes through the purification procedure.

The *A. baumannii* Ribosome Has Unique Structural Features, Including Around the Exit of the Polypeptide Tunnel and the Subunit Interface

Structural differences in bacterial ribosomes may be exploited to design drugs that have improved activity against pathogenic species or that avoid harmful side effects resulting from disturbance of the gut microbiota (Dethlefsen et al., 2008). To identify unique structural features in the *A. baumannii* ribosome, the structure was compared with that of ribosomes from other bacteria, including *E. coli* (PDB: 4YBB and PDB: 5MDZ) (James et al., 2016; Noeske et al., 2015), *S. aureus* (PDB: 5LI0) (Khusainov et al., 2016), and *T. thermophilus* (PDB: 5E81) (Rozov et al., 2016). Insertions, deletions, and differences in the fold of all modeled ribosomal proteins and rRNA in the *A. baumannii* ribosome compared with *E. coli* were identified (Table S3). The overall architecture of this ribosome is broadly similar to that of other bacterial ribosomes; in particular, the regions around the catalytic peptidyl transferase center and decoding center are structurally conserved. Differences were mostly located on the solvent-facing portions of the subunits, as well as around the periphery of the subunit interface (Figures 2A and 2B).

Hotspots of structural differences were identified around the exit of the polypeptide tunnel on the solvent-exposed face of the 50S subunit, specifically in proteins uL22, uL23, uL24, and bL32, and in the 23S rRNA helices H6, H7, and H9 (Figure 2C).

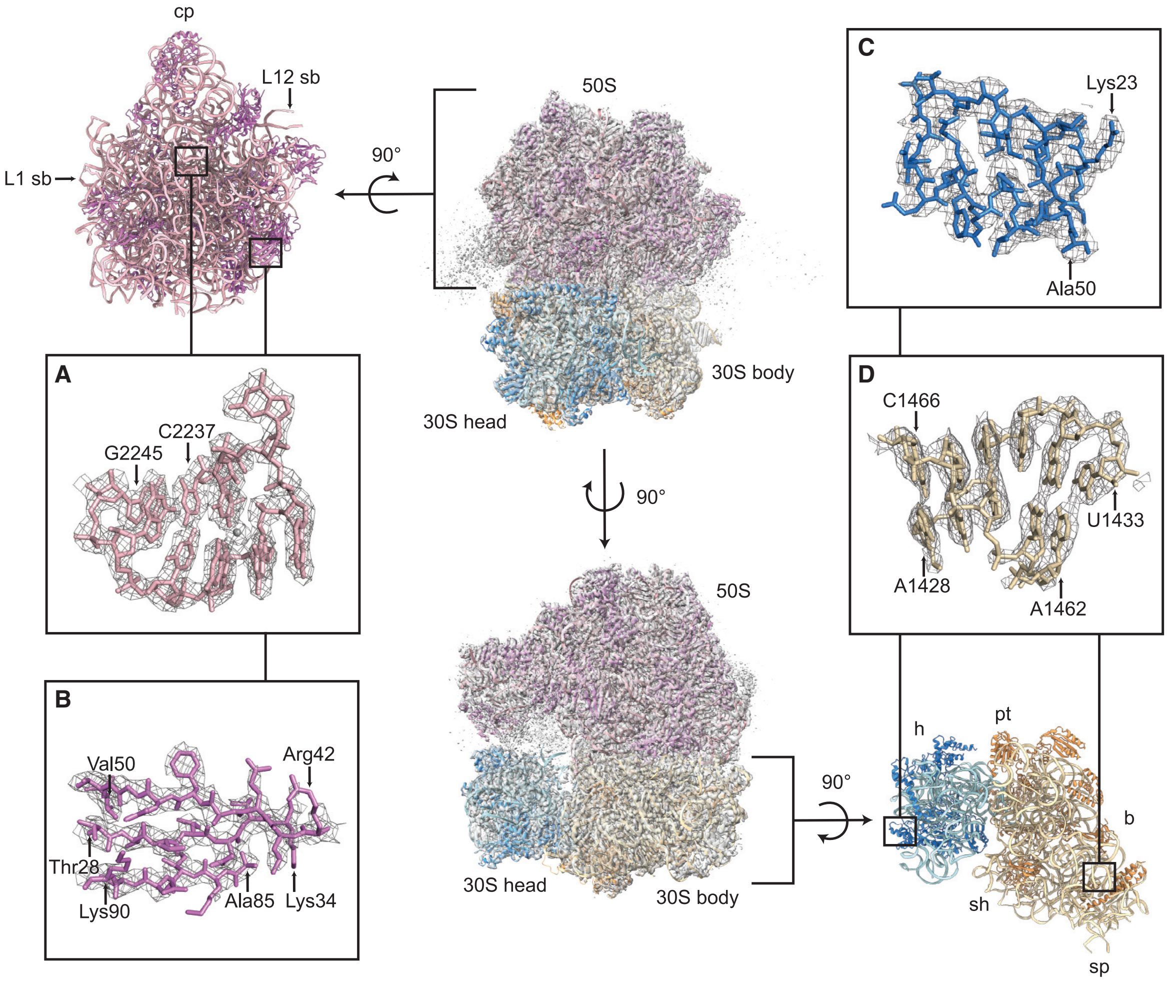

Figure 1. Structure of the 70S Ribosome from *A. baumannii*

Center: two views of the *A. baumannii* ribosome-amikacin complex with atomic models of the 50S (rRNAs light pink, proteins dark pink), 30S body (rRNA light brown, proteins dark brown) and 30S head (rRNA light blue, proteins dark blue), and corresponding EM densities (gray volume). These three models were refined separately in the three multibody reconstructions (after sharpening and masking), and the models and maps are superimposed. Top left: atomic model of the 50S subunit showing key structural elements (cp, central protuberance; L1 sb, L1 stalk base; L12 sb, L12 stalk base). Bottom right: atomic model of the 30S subunit showing key structural elements (h, head; pt, platform; b, body; sp, spur; sh, shoulder).
(A) EM density (gray mesh) and atomic model of the 23S rRNA P loop C2237–G2245.
(B) EM density (gray mesh) and atomic model of a β sheet in protein bL19: Thr28–Lys34, Arg42–Val50, Ala85–Lys90.
(C) EM density (gray mesh) and atomic model of a helix-turn-helix in protein uS14: Lys23–Ala50.
(D) EM density (gray mesh) and atomic model of part of the 16S rRNA helix h44: A1428–U1433, A1462–C1466.
See also Table S2.

This site is where ribosome-associated factors dock, such as the molecular chaperone trigger factor (Deuerling et al., 2019), the signal recognition particle, and the Sec translocon (Jomaa et al., 2016). For example, the 23S rRNA helix H6, situated near uL23 at the edge of the polypeptide tunnel exit, takes up a different fold in *A. baumannii* compared with *E. coli*, *S. aureus*, and *T. thermophilus* (Figure 3A). The fold of H6 is consistent across multiple *E. coli* structures, no matter the structural technique or ribosome buffer used, so this difference in fold is attributed to species specificity (Figure 3B). Note that the fold of the nearby β-hairpin loop of uL23, although clearly different between the different species (Figure 3A), does not always maintain the same fold in different *E. coli* structures (Figure 3B). This makes it more difficult to assign as a species-specific difference with as high a confidence as H6, as different buffer conditions or crystal contacts could be playing a role. Clusters of differences were also identified around the periphery of the subunit interface, specifically in the 23S rRNA helices H58 and H63, the 16S rRNA helices h8 and h44, and protein uL14 (Figure 2D). The consensus reconstruction (i.e., the EM map determined without using multibody refinement) was used to check the validity of the multibody models in these regions, because models fitted to multibody maps can be difficult to interpret at the body interfaces (Nakane et al., 2018). H63 is shorter in *S. aureus* and *T. thermophilus* than in *E. coli* or *A. baumannii*, but takes up a slightly different conformation in *E. coli* compared with *A. baumannii*

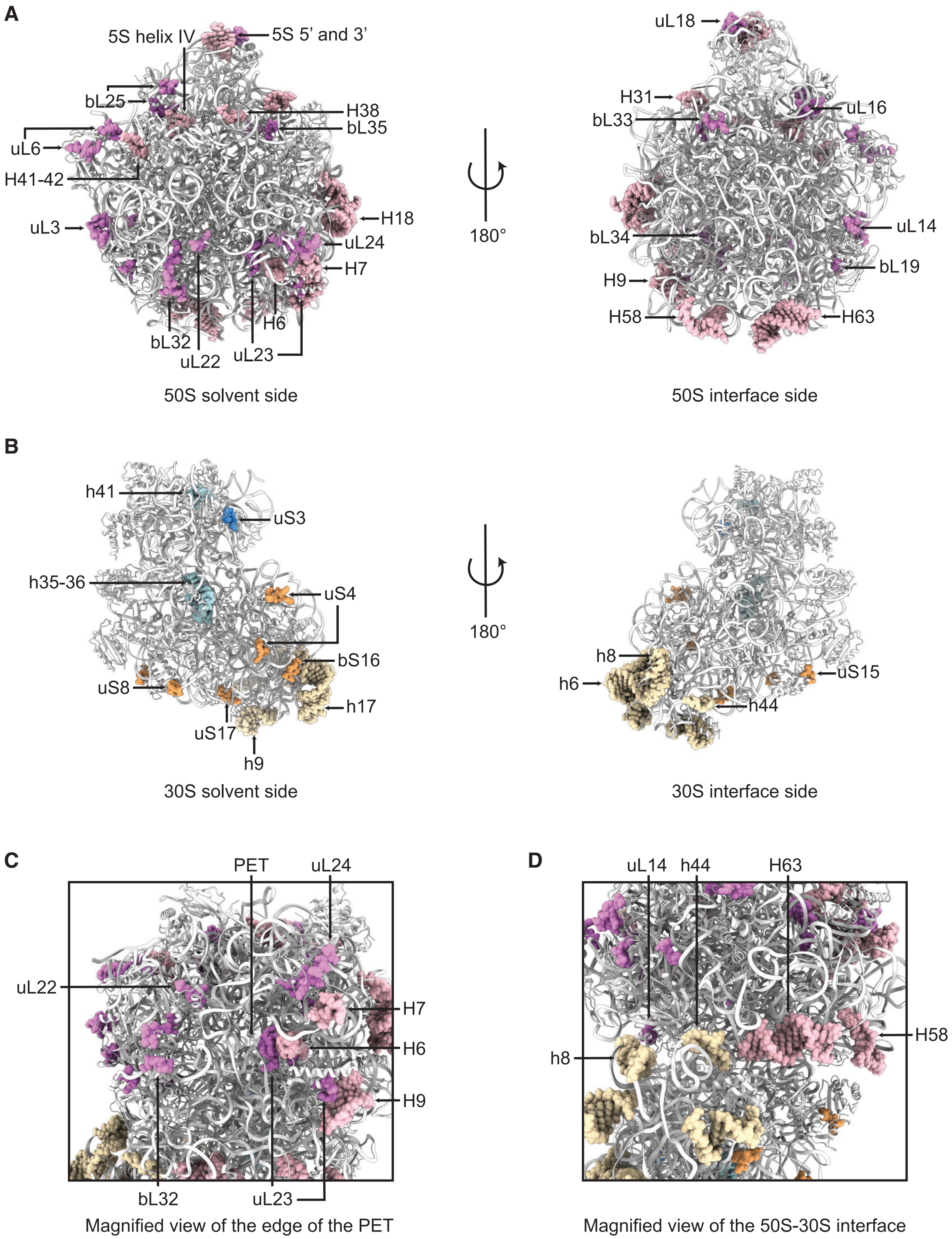

Figure 2. Unique Structural Features of the *A. baumannii* Ribosome

Insertions, deletions, and differences in the fold of loops and secondary structures of the ribosomal proteins and rRNA in the *A. baumannii* ribosome compared with *E. coli* (PDB: 5MDZ and 4YBB).

(A) Two views of the atomic model of the 50S subunit (white cartoon), with unique features highlighted (rRNA light pink spheres, protein dark pink spheres).

(B) Two views of the atomic model of the 30S subunit (white cartoon), with unique features highlighted (30S body rRNA light brown spheres, protein dark brown spheres; 30S head rRNA light blue spheres, protein dark blue spheres).

(C) Magnified view of unique features around the edge of the PET (polypeptide exit tunnel).

(D) Magnified view of unique features around the subunit interface.

The structure of the *A. baumannii* ribosome-amikacin complex is used, but all highlighted differences hold true for the *A. baumannii* ribosome-tigecycline complex. See also Table S3.

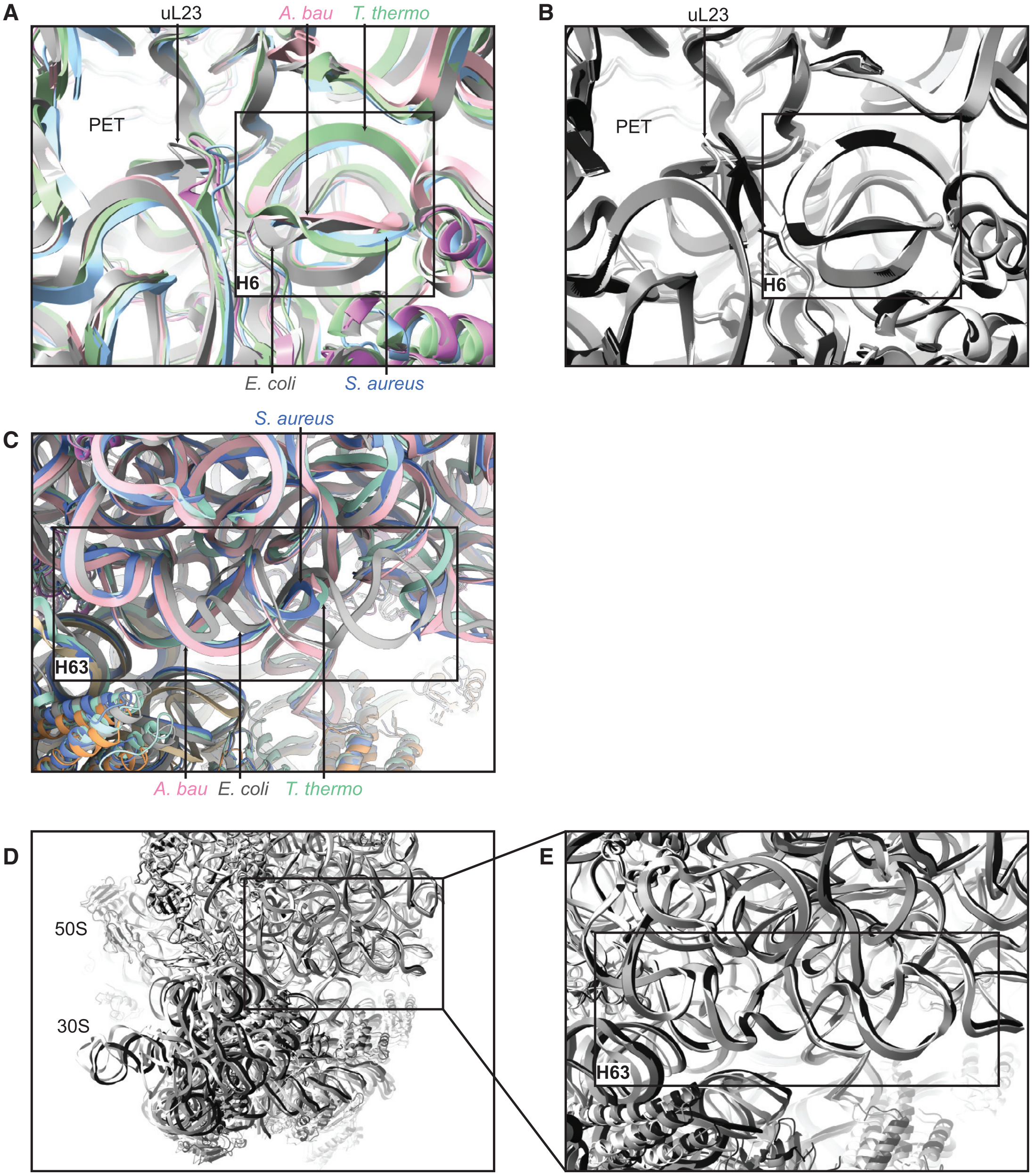

Figure 3. Unique Structural Features in the 23S rRNA Helices H6 and H63

(A) H6 of *A. baumannii* (pink) takes a different conformation compared with H6 of *E. coli* (gray, PDB: 4YBB), *S. aureus* (blue, PDB: 5LI0), and *T. thermophilus* (green, PDB: 5E81).

(B) H6 maintains the same conformation across various *E. coli* ribosome structures, as shown here in a crystal structure (light gray, PDB: 4YBB ribosome I; mid-gray, PDB: 4YBB ribosome II) and an EM structure (dark gray, PDB: 5MDZ), unlike the nearby β-hairpin loop of uL23, which shows some variation.

(C) H63 of *A. baumannii* (pink) takes a different conformation than H63 of *E. coli* (gray, PDB: 4YBB) and is longer than in *S. aureus* (blue, PDB: 5LI0) and *T. thermophilus* (green, PDB: 5E81).

(D) *E. coli* ribosome models aligned on the 50S ribosomal subunit, representing a range of rotation states of the 30S ribosomal subunit. Empty ribosome in an intermediate rotated state (light gray, PDB: 4YBB ribosome I), empty ribosome in non-rotated state (mid-gray, PDB: 4YBB ribosome II), and ribosome with A-site and P-site tRNA (dark gray, PDB: 5MDZ).

(E) H63 in these three *E. coli* ribosome models. Despite the difference in intersubunit rotation states, the conformation of H63 remains similar across the three models.

The structure of the *A. baumannii* ribosome-amikacin complex is used, but the structures of all highlighted regions hold true for the *A. baumannii* ribosome-tigecycline complex. PET, polypeptide exit tunnel.

CellPress
OPEN ACCESS

(Figure 3C). Contacts at the interface keep the ribosome intact and are important for the dynamic processes involved in translocation (Liu and Fredrick, 2016) and some antibiotics, such as neomycin and thermorubin, inhibit translation by perturbing these intersubunit bridges (Bulkley et al., 2012; Wang et al., 2012). To confirm the validity of the unique structural features in H63 of *A. baumannii*, as well as the other identified features near the subunit interface, we compared these helices and proteins with those in *E. coli* ribosome structures exhibiting a range of 50S-30S rotation states: the two ribosome conformations in PDB: 4YBB and the ribosome from PDB: 5MDZ (Figure 3D). The conformations of these helices and proteins remained consistent across these three *E. coli* ribosome structures, and hence were all different from the corresponding conformations in the *A. baumannii* ribosome (Figure 3E shows this for H63). This strongly suggests that the unique structural features identified near the subunit interface truly are species-specific differences, rather than differences arising from different ribosome rotation states.

The structure of the ribosome from *A. baumannii* was very recently solved in empty and tRNA-bound states, and the authors identified three unique structural features of this ribosome (Morgan et al., 2020). First, H18 was significantly shorter than in other bacterial ribosomes, which is confirmed in our structure (Figure 4A). The authors also identified a conformational difference in H58, which forms a straight helix, without bending to contact H54/H55 as it does in other ribosomes. However, in our structure, although H58 is longer and has a slightly different conformation in *A. baumannii* compared with *E. coli*, the helix still follows an overall similar path, forming the contact with H54/H55 (Figure 4B). Finally, the authors found that H69 bends toward the 50S subunit instead of forming intersubunit bridge B2a/d with h44 of the 30S subunit, much the same as seen in the aminoglycoside-resistant *P. aeruginosa* ribosome structure (Halfon et al., 2019a), and they suggested this could contribute toward low aminoglycoside susceptibility. However, the strain of *A. baumannii* used in their study (AB0057) is reported to be susceptible to aminoglycosides amikacin and tobramycin (Adams et al., 2008), and in our structure, H69 forms contacts with h44 as seen in other bacterial ribosomes (Figure 4C), suggesting a more complex and subtle relationship between the conformation of H69 and aminoglycoside resistance. The cause of the differences in H58 and H69 in these two structures is unclear. Indeed, there are no sequence differences in the stem-loops or in the proteins in the immediate vicinity that might explain any change in conformation. However, similar purification procedures and final ribosome buffers were used when preparing the two samples, so it is unlikely that these differences are artifactual. Note that these differences are not due to modeling errors, as the density is quite clear for both helices in both structures (Figure S6). Discrepancies between these two structures imply that even ribosomes from different strains of the same bacterial species may have unique structural features, and further study could help us to understand divergent antibiotic susceptibilities in different strains.

The Interaction of Antibiotics Amikacin and Tigecycline with the *A. baumannii* Ribosome

Additional cryo-EM density is present for amikacin and tigecycline in the amikacin-ribosome and tigecycline-ribosome cryo-EM reconstructions, respectively (Figure 5). In this section, *A. baumannii* rRNA nucleotide numbering will be followed by the identity of the corresponding *E. coli* nucleotide in parentheses. Aminoglycosides are known to impede the translocation of the mRNA-tRNA complex through the ribosome (Tsai et al., 2013), inhibit ribosome recycling (Borovinskaya et al., 2007), and promote translational misreading (Wilson, 2009). Like other aminoglycosides (Carter et al., 2000; Ogle et al., 2001), amikacin binds within an internal loop in h44 of the 16S rRNA at the A site and sterically overlaps with nucleotides A1489 (A1492) and A1490 (A1493), promoting an alternate conformation where these nucleotides are flipped out into the decoding center (Figure 5C). These nucleotides usually probe the minor groove of the codon-anticodon duplex in the A site, and hence distinguish cognate from non-cognate tRNAs. The stabilization of this flipped-out conformation even in the presence of non-cognate tRNA is a plausible mechanism for aminoglycoside-induced misreading (Magnet and Blanchard, 2005). Nearby, A1902 (A1913) of the 23S rRNA H69 moves toward the tRNA binding site, and the phosphate of A1490 (A1493) moves in toward to center of h44, away from the decoding center (Figure 5C). These movements fit with an alternative model proposing that aminoglycoside binding promotes misreading by inducing local changes in h44 and H69, which relax the constraints of the decoding pocket and otherwise compensate for energetically unfavorable non-cognate tRNA-mRNA interactions (Demeshkina et al., 2012). It may be that the concerted effect of both these mechanisms ultimately drives misreading.

Tetracyclines inhibit translation elongation by binding to the 30S ribosomal subunit and interfering with the delivery of A-site tRNA (Brodersen et al., 2000; Pioletti et al., 2001). Tigecycline, a third-generation tetracycline derivative, targets the head of the 30S subunit to overlap with the A site in a way similar to that of tetracycline. It interacts with the phosphate backbone of h34 of the 16S rRNA through coordination between polar groups of rings B and C and a magnesium ion (Mg-1), and with the 16S rRNA nucleotides C1051 (C1054) and A1193 (A1196) through a stacking interaction of its 9-t-butylglycylamido moiety (Figure 5E), a group not present in tetracycline. Also, the sugar ring of C1192 (C1195) is in a position to form polar interactions with the amide of ring A of tigecycline. These interactions are similar to those seen in other structures (Figure 6) (Cocozaki et al., 2016; Jenner et al., 2013; Schedlbauer et al., 2015); however, there are a few noteworthy differences. First the nature of the stacking interaction of tigecycline with C1051 (C1054) varies slightly in the different structures. This interaction is thought to account for tigecycline's increased ability to interfere with A-site tRNA binding as well as its increased binding affinity compared with tetracycline (Jenner et al., 2013), and may also hinder access of the ribosomal protection protein TetM, likely explaining tigecycline's ability to evade TetM-mediated resistance (Jenner et al., 2013; Schedlbauer et al., 2015). In the *A. baumannii* 70S ribosome-tigecycline and the *T. thermophilus* 30S-tigecycline complexes, the base of C1051 (C1054) appears to form a pi-pi stacking interaction with ring D of tigecycline (Figures 6A and 6C), whereas it appears to stack with the amide of the 9-t-butylglycylamido moiety in the *T. thermophilus* 70S ribosome-tigecycline and *E. coli* 70S ribosome-tigecycline complexes (Figures 6B and 6D). Furthermore, although the density

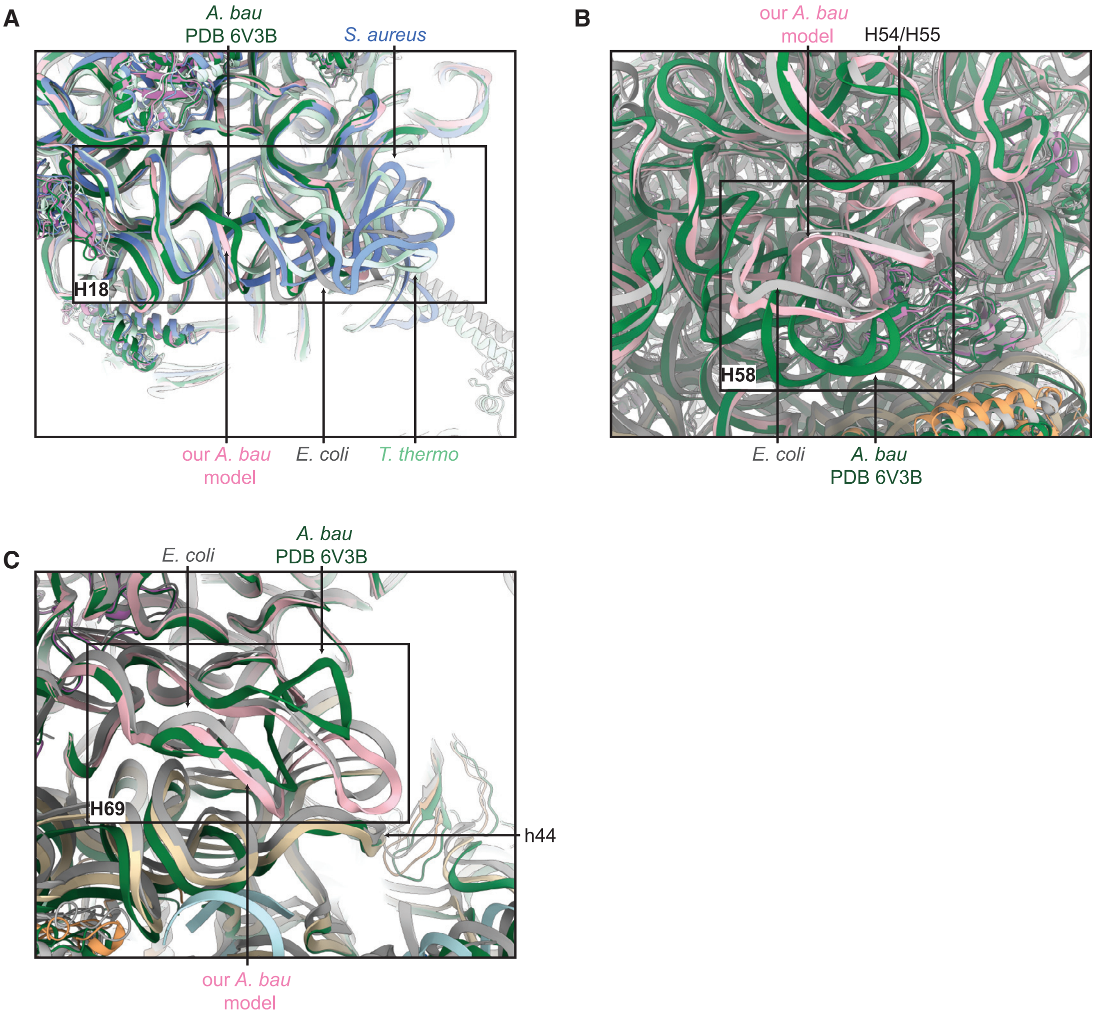

Figure 4. Structural Comparison of the Ribosome from Two Different Strains of *A. baumannii*

(A) H18 takes up different conformations in *A. baumannii* (pink, our model; dark green, PDB: 6V3B), *E. coli* (gray, PDB: 4YBB), *S. aureus* (blue, PDB: 5LI0), and *T. thermophilus* (light green, PDB: 5E81).

(B) H58 bends to interact with H54/H55 in our model of the *A. baumannii* ribosome (pink, strain ATCC 19606) and the *E. coli* ribosome (gray, PDB: 4YBB), but not in a different *A. baumannii* ribosome (dark green, strain AB0057, PDB: 6V3B).

(C) H69 reaches toward the 30S subunit to interact with h44 in our model of the *A. baumannii* ribosome (pink, strain ATCC 19606) and the *E. coli* ribosome (gray, PDB: 4YBB), but instead bends back toward the 50S subunit in a different *A. baumannii* ribosome (dark green, strain AB0057, PDB: 6V3B).

The structure of the *A. baumannii* ribosome-amikacin complex is used to describe the ATCC 19606 *A. baumannii* ribosome, but the structures of all highlighted regions hold true for the *A. baumannii* ribosome-tigecycline complex.

See also Figure S6.

of the 9-t-butylglycylamido moiety is not strong in our data, it appears to adopt an extended conformation, similar to that seen when tigecycline is bound to the *T. thermophilus* 30S subunit, rather than a bent conformation as seen when bound to the whole *T. thermophilus* 70S ribosome. This bent conformation has previously been suggested to help accommodate a "closed" conformation of h18, which occurs when the 30S head and shoulder rotate inwards toward the decoding center (Schedlbauer et al., 2015). This is supported by a comparison of the structures of tigecycline bound to the *T. thermophilus* 70S ribosome and the *T. thermophilus* 30S ribosomal subunit, where the movement of h18 away from the tigecycline site in the latter correlates with an extended conformation of tigecycline's 9-t-butylglycylamido moiety (Figures 6B and 6C). In the *A. baumannii* ribosome-tigecycline structure presented here, h18 is even farther away from the tigecycline site, and the 9-t-butylglycylamido moiety adopts an extended conformation, following the expected trend (Figure 6A). (Note that the consensus map was used to confirm the relative proximity of these features, because the tigecycline site forms part of the 30S head, whereas h18 is part of the 30S body.) It should be noted that the 9-t-butylglycylamido moiety adopts a bent conformation in the *E. coli* 70S ribosome structure, despite h18 being far from the tigecycline binding site, which appears to contradict this trend (Figure 6D). However, the density corresponding to this moiety is not well defined, so it is not clear whether it in fact

CellPress
OPEN ACCESS

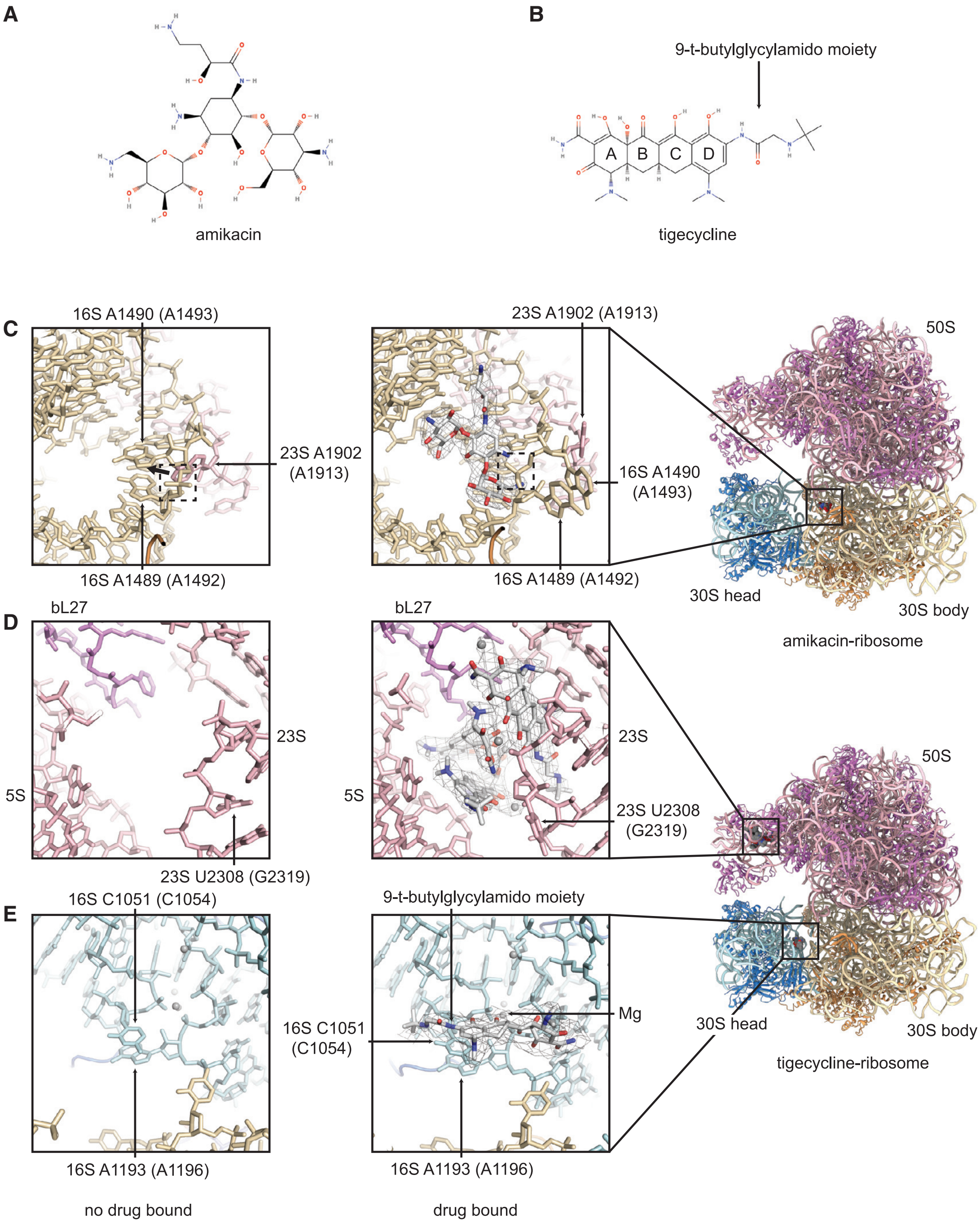

Figure 5. Interactions of Amikacin and Tigecycline with the *A. baumannii* Ribosome

(A) Structural formula of amikacin.

(B) Structural formula of tigecycline. Both drawn in MolView.

(C) The aminoglycoside binding site with amikacin bound (right), drug shown as an atomic model (white) with carved EM density (gray mesh). The tigecycline-ribosome structure, left, shows this site with no amikacin bound. Nucleotides A1489 and A1490 of the 16S rRNA and A1902 of the 23S rRNA, and the phosphate bridging A1489 and A1490, which change conformation upon drug binding, are highlighted.

(D) A secondary tigecycline binding site with tigecycline bound (right), drug shown as an atomic model (white) with carved EM density (gray mesh). The amikacin-ribosome structure, left, shows the site with no tigecycline bound. Nucleotide U2308 of the 23S rRNA, which changes conformation upon drug binding, is highlighted.

(E) The primary tigecycline binding site with tigecycline bound (right), drug shown as an atomic model (white) with carved EM density (gray mesh). The amikacin-ribosome structure, left, shows the site with no tigecycline bound. Nucleotides C1051 and A1193 of the 16S rRNA, along with a magnesium ion, which all interact with the drug, are highlighted.

E. coli numbering is shown in parentheses.

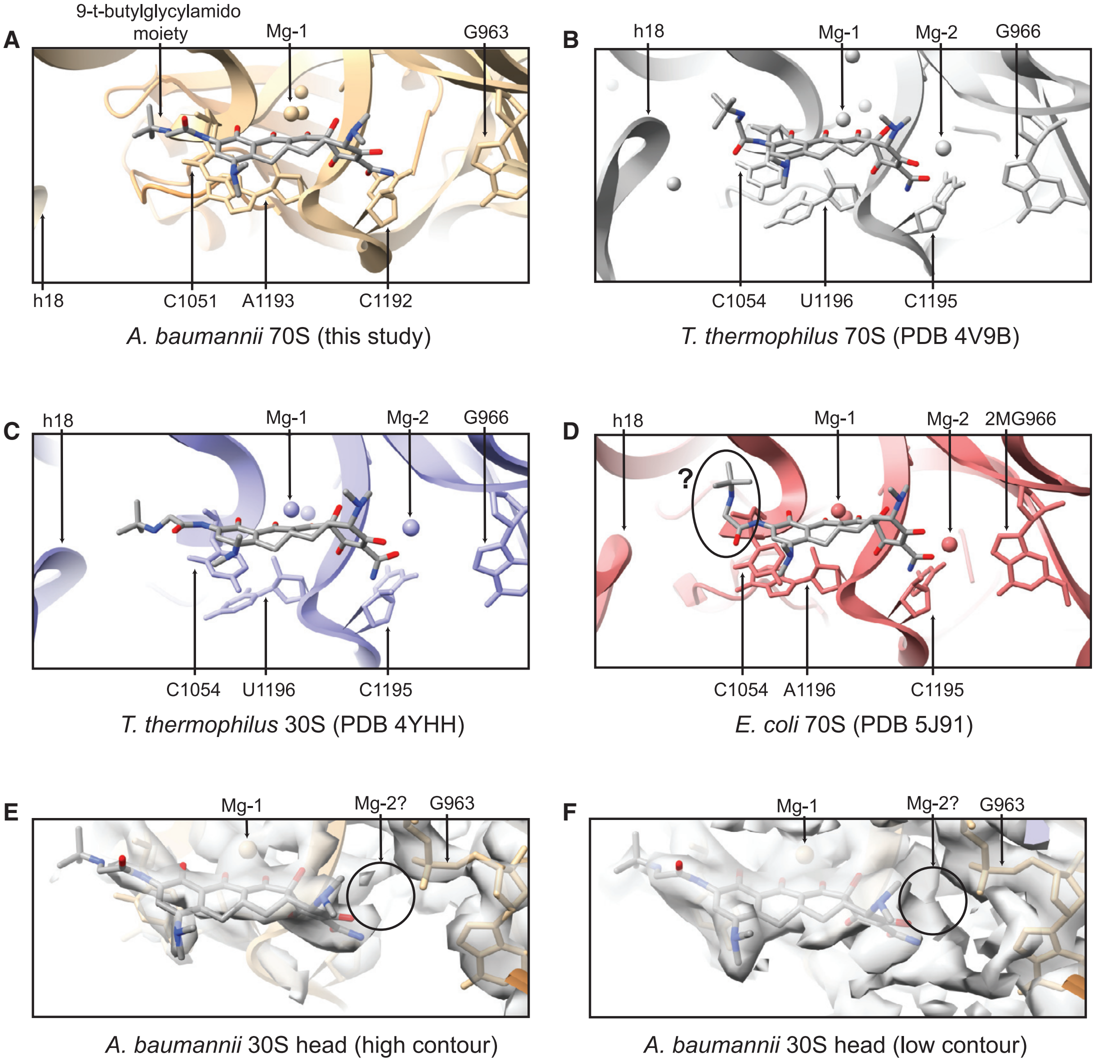

Figure 6. The Primary Tigecycline Binding Site in Ribosomes and Ribosome Subunits of Different Bacteria

(A–D) Atomic models of the primary tigecycline binding site in ribosomes and ribosome subunits of different bacteria. The nature of tigecycline binding is broadly similar across the structures, with some differences in the stacking interaction of tigecycline with C1051 (C1054) of 16S rRNA, the conformation of the 9-t-butylglycylamido moiety, and the coordination of a second magnesium ion. (A) Atomic model of tigecycline (gray) bound to the 70S of the *A. baumannii* ribosome (brown). The 30S head model and h18 from the 30S body model are shown. The consensus map was used to confirm the relative proximity of these features. (B) Atomic model of tigecycline (gray) bound to the 70S *T. thermophilus* ribosome (gray, PDB: 4V9B). (C) Atomic model of tigecycline (gray) bound to the 30S *T. thermophilus* ribosomal subunit (blue, PDB: 4YHH). (D) Atomic model of tigecycline (gray) bound to the 70S *E. coli* ribosome (red, PDB: 5J91). The density is not strong enough to support either an extended or a bent conformation of the 9-t-butylglycylamido moiety, as indicated by the question mark.

(E) EM density of the 30S head of the *A. baumannii* ribosome-tigecycline complex at the primary tigecycline site, high-contour level.

(F) EM density of the 30S head of the *A. baumannii* ribosome-tigecycline complex at the primary tigecycline site, low-contour level. It is difficult to discern possible magnesium ion density (Mg-2) from noise.

adopts a bent, rather than an extended, conformation (Cocozaki et al., 2016). In addition, all previous tigecycline-ribosome structures place a magnesium ion (Mg-2) that coordinates ring A of tigecycline to the phosphate of G963 (2N-methyl-G966). Through examination of the exact location of this ion, in the *T. thermophilus* 70S-tigecycline structure this coordination appears to occur primarily through ring A's hydroxyl oxygen, in the *T. thermophilus* 30S-tigecycline structure primarily through the ring's amide oxygen, and in the *E. coli* 70S-tigecycline structure there is an even mixture of the two (Figure 6). However, there is no density for a second magnesium ion in the *A. baumannii* ribosome-tigecycline map at high contour levels (Figure 6E). At lower contour levels there is some density present in this region, though at these levels, signal is difficult to discern from noise (Figure 6F). This is in contrast to the magnesium ion in site 1 (Mg-1), which has strong and clearly defined EM density.

Additional density for tigecycline was also seen in the 50S at the central protuberance. Here, three tigecycline molecules bind in a cavity between the 23S rRNA, the 5S rRNA, and protein bL27. This is accommodated by a significant conformational

CellPress
OPEN ACCESS

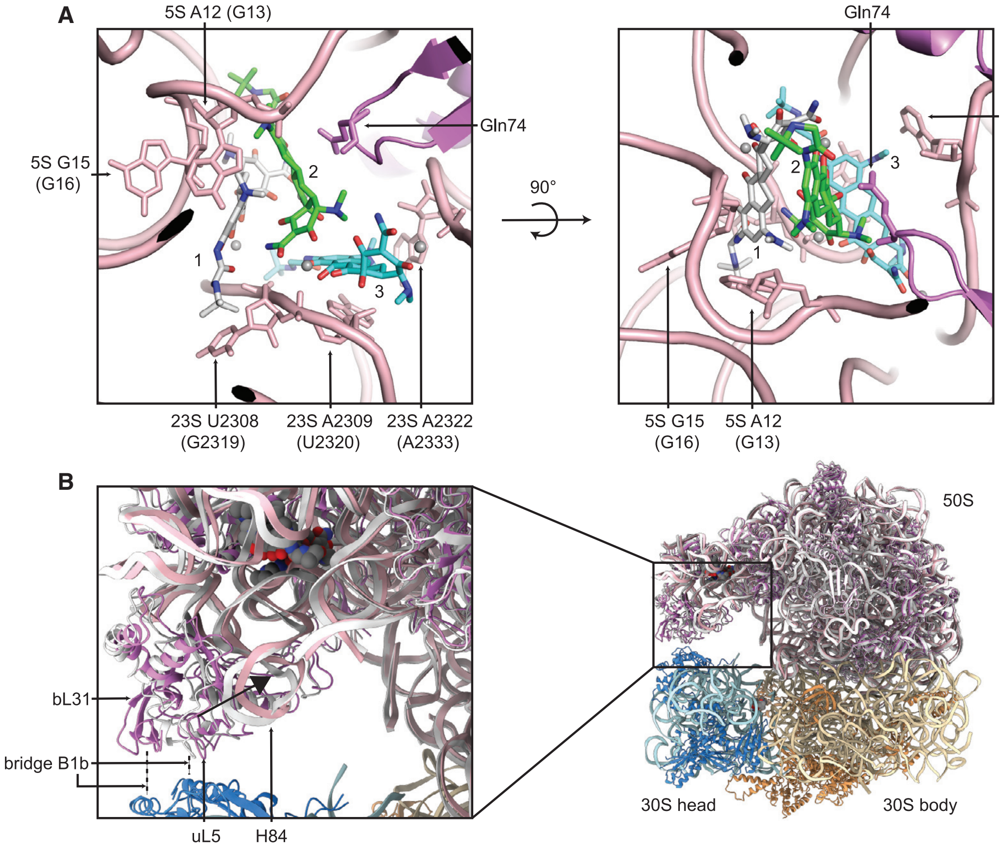

Figure 7. A Secondary Tigecycline Binding Site at the 50S Central Protuberance

(A) Two views of the secondary binding site in the *A. baumannii* ribosome-tigecycline structure. The three tigecycline molecules are labeled 1 (white), 2 (green), and 3 (cyan). The 23S rRNA nucleotides U2308, U2309, and A2322; the 5S rRNA nucleotides A12 and G15; and the bL27 residue Gln74, which interact with the drug molecules, are labeled. Magnesium ions are shown as gray spheres.

(B) Changes in the conformation of the central protuberance and intersubunit bridge B1b upon tigecycline binding at this secondary site. The atomic model of the tigecycline-bound ribosome (50S pink, 30S body brown, 30S head blue) is overlaid with the atomic model of the amikacin-bound 50S, which has no tigecycline bound (white). H84 and uL5 undergo a shift upon tigecycline binding, and bL31 becomes partially resolved in the density.

E. coli numbering is shown in parentheses.

See also Figure S7.

change of the surrounding rRNA, with a particularly large movement by U2308 (G2319) of the 23S rRNA, which flips out to interact with the 9-t-butylglycylamido moiety of one of the molecules (Figure 5D). The three molecules interact with one another through stacking interactions and through bridging magnesium ions, as well as with the backbone and bases of the surrounding rRNA and protein bL27 (Figure 7A, the three molecules labeled 1, 2, and 3). Interactions between these ligands and the surrounding ribosome were calculated using Arpeggio (Jubb et al., 2017). From the 5S rRNA, the base of A12 (G13) forms a carbon-pi interaction with a methyl group of the amine of ring D of tigecycline 1, and the 2′-OH of G15 (G16) forms a donor-pi interaction with ring D of tigecycline 1. From the 23S rRNA, a carbonyl and a sugar ring oxygen of U2308 (G2319) form polar contacts with the 9-t-butylglycylamido moiety of tigecycline 1, the base of A2309 (U2320) forms a pi-pi stacking interaction with ring D of tigecycline 3, and the base of A2322 (A2333) forms a carbon-pi interaction with a methyl group of the amine of ring D of tigecycline 3. Finally, the main-chain carbonyl and Cγ of Gln74 of bL27 form van der Waals contacts with the amide of ring A and ring B of tigecycline 2. Additional interactions between the tigecycline molecules and the surrounding phosphate rRNA backbone are facilitated by coordination of magnesium ions, most clearly seen by the ion that bridges the phosphate of 23S A2309 (U2320) with oxygen atoms of ring A of tigecycline 2 and rings B and C of tigecycline 3. These interactions are summarized in 2D in Figures S7A–S7C. Overall, the presence of these tigecycline molecules promotes a series of interactions bridging the 23S and 5S rRNAs, with contributions from bL27.

To investigate the possibility of tigecycline binding to this secondary site in other bacteria, the structure of this site in the *A. baumannii* ribosome-amikacin model was compared with that in ribosome structures from *E. coli*, *S. aureus*, and *T. thermophilus*, so that the site is compared in its empty state (Figure S7). The sites in *A. baumannii* and *E. coli* are very similar (Figure S7D), with the most noticeable differences found in U2308 (G2319) of the 23S rRNA, which takes up slightly different conformations in the two structures. However, given that this base dramatically changes conformation on tigecycline binding anyway, it is difficult to conclude whether this difference would have an impact

on binding. Another obvious difference is the identity of residue 74 of bL27, which is glutamine in *A. baumannii* but proline in *E. coli*. However, in *A. baumannii* this residue appears only to form van der Waals contacts with tigecycline through its main chain and Cγ, and these contacts could probably still be made with a proline residue. The secondary tigecycline site differs much more greatly in *S. aureus* and *T. thermophilus* (Figure S7E). In these structures, the surrounding rRNA and the loop in bL27 take up different folds compared with their equivalents in *A. baumannii*. Overall, this crude analysis suggests that tigecycline might be able to bind to this site in *E. coli*, but is less likely to bind in *S. aureus* and *T. thermophilus*. However, experimental evidence is needed to confirm or refute this hypothesis.

These interactions appear to have a long-range effect beyond the binding pocket, with H84 of the 23S rRNA pulled toward the binding site, along with uL5, which is pulled away from its interaction with the 30S where it normally forms part of bridge B1b (Figure 7B). Protein bL31, which also forms part of bridge B1b, is partly resolved in this tigecycline-bound structure but much less well resolved in the amikacin-bound structure, so it appears that these conformational shifts make bL31 more rigid.

It could be that this additional tigecycline binding at the central protuberance of the 50S is an artifact from incubation of the ribosome with excess drug. Indeed, the stacking of three separate drug molecules is unusual, and no unambiguous density for tigecycline in this site was seen in the other two tigecycline-70S ribosome structures (Cocozaki et al., 2016; Jenner et al., 2013), which are both X-ray crystal structures involving ribosomes from different species. However, it should be noted that none of the previously reported secondary binding sites for tetracycline within the 30S subunit were occupied in the EM density of our structure (Brodersen et al., 2000; Pioletti et al., 2001). In our density, the site at the central protuberance was the only place where density for tigecycline was seen, other than the reported primary site, suggesting that tigecycline binds at least moderately tightly to this secondary site, and it is possible that the rearrangement of bridge B1b caused by the binding of tigecycline here could affect the stability of the ribosome or the dynamics of translation. Whether tigecycline binding at this site contributes a secondary mode of action for the drug beyond inhibition of A-site tRNA delivery is currently unknown.

Accelerating Antibiotic Discovery Informed by Ribosome Structures

In summary, we have presented the structure of the *A. baumannii* ribosome in complex with clinically relevant antibiotics. Unique structural features were identified, for example, regions around the exit of the polypeptide tunnel, which could be targeted to interfere with ribosome-associated factor binding and hence nascent chain folding or targeting, and regions around the subunit interface, which could be targeted to destabilize ribosome integrity or dynamics. Furthermore, the interactions of the antibiotics amikacin and tigecycline with the *A. baumannii* ribosome were elucidated, revealing changes in ribosome conformations and, in the case of tigecycline, a putative additional binding site.

As in this study, previously determined structures of drug-ribosome complexes tend to involve empty ribosomes (Cocozaki et al., 2016), or sometimes ribosomes with tRNA and mRNA bound *in vitro* (Jenner et al., 2013). These structures provide an important starting point to understand the action of ribosome-targeting antibiotics in different bacteria and may be used to aid the design of new drugs. They also pave the way for structural studies on more complex systems in which ribosomes are stalled or inhibited mid-translation by the drug in question to gain snapshots of antibiotics ''in action,'' trapping the ribosome in particular conformational states. Some previous structures have already been determined using this approach, such as the erythromycin-bound ErmBL- and ErmCL-stalled bacterial ribosomes (Arenz et al., 2014a, 2014b) and the PF846-stalled human ribosome (Li et al., 2019). Building a repertoire of structures of drug-bound ribosomes that are empty, filled with tRNA and mRNA, or stalled in particular conformational states, and which are isolated from a variety of bacterial species and strains, will provide a strong platform for the design of new drugs with improved activity against specific species or strains of bacteria and which inhibit different stages of translation. Only by accelerating development of new antibiotics will we be able to successfully treat increasingly drug-resistant infections in the future, including those caused by *A. baumannii*.

STAR★METHODS

Detailed methods are provided in the online version of this paper and include the following:

- KEY RESOURCES TABLE
- RESOURCE AVAILABILITY
  - Lead Contact
  - Materials Availability
  - Data and Code Availability
- EXPERIMENTAL MODEL AND SUBJECT DETAILS
- METHOD DETAILS
  - 70S Ribosome Purification
  - CryoEM
  - Image Processing
  - Atomic Model Building and Refinement
  - Figures and Model Analysis
- QUANTIFICATION AND STATISTICAL ANALYSIS

SUPPLEMENTAL INFORMATION

Supplemental Information can be found online at https://doi.org/10.1016/j.str.2020.08.004.

ACKNOWLEDGMENTS

This work was supported by a Wellcome Trust 4-year PhD studentship to D.N. (203743/Z/16/Z) and BBSRC grant to AJO (BB/R004048/1). All cryo-EM was performed at the Astbury Biostructure Laboratory (ABSL), which was funded by the University of Leeds and the Wellcome Trust (108466/Z/15/Z). We thank ABSL for helping with cryo-EM data collection and Drs. Matthew Iadanza and Shaun Rawson for assistance with Python scripting.

AUTHOR CONTRIBUTIONS

D.N. carried out experiments and analyzed the data. D.N., T.A.E., A.J.O., and N.A.R. conceived and directed the project. D.N., T.A.E., A.J.O., and N.A.R. wrote the manuscript.

DECLARATION OF INTERESTS

The authors declare no competing interests.

Received: April 9, 2020
Revised: July 6, 2020
Accepted: August 7, 2020
Published: August 27, 2020

REFERENCES

Adams, M.D., Goglin, K., Molyneaux, N., Hujer, K.M., Lavender, H., Jamison, J.J., MacDonald, I.J., Martin, K.M., Russo, T., Campagnari, A.A., et al. (2008). Comparative genome sequence analysis of multidrug-resistant *Acinetobacter baumannii*. J. Bacteriol. *190*, 8053–8064.

Adams, P.D., Afonine, P.V., Bunkoczi, G., Chen, V.B., Davis, I.W., Echols, N., Headd, J.J., Hung, L.W., Kapral, G.J., Grosse-Kunstleve, R.W., et al. (2010). PHENIX: a comprehensive Python-based system for macromolecular structure solution. Acta Crystallogr. D Biol. Crystallogr. *66*, 213–221.

Arenz, S., Meydan, S., Starosta, A.L., Berninghausen, O., Beckmann, R., Vazquez-Laslop, N., and Wilson, D.N. (2014a). Drug sensing by the ribosome induces translational arrest via active site perturbation. Mol. Cell *56*, 446–452.

Arenz, S., Ramu, H., Gupta, P., Berninghausen, O., Beckmann, R., Vazquez-Laslop, N., Mankin, A.S., and Wilson, D.N. (2014b). Molecular basis for erythromycin-dependent ribosome stalling during translation of the ErmBL leader peptide. Nat. Commun. *5*, 3501.

Asif, M., Alvi, I.A., and Rehman, S.U. (2018). Insight into *Acinetobacter baumannii*: pathogenesis, global resistance, mechanisms of resistance, treatment options, and alternative modalities. Infect. Drug Resist. *11*, 1249–1260.

Ban, N., Nissen, P., Hansen, J., Moore, P.B., and Steitz, T.A. (2000). The complete atomic structure of the large ribosomal subunit at 2.4 A resolution. Science *289*, 905–920.

Baron, S., Hadjadj, L., Rolain, J.M., and Olaitan, A.O. (2016). Molecular mechanisms of polymyxin resistance: knowns and unknowns. Int. J. Antimicrob. Agents *48*, 583–591.

Berman, H., Henrick, K., and Nakamura, H. (2003). Announcing the worldwide protein data bank. Nat. Struct. Biol. *10*, 980.

Borovinskaya, M.A., Pai, R.D., Zhang, W., Schuwirth, B.S., Holton, J.M., Hirokawa, G., Kaji, H., Kaji, A., and Cate, J.H. (2007). Structural basis for aminoglycoside inhibition of bacterial ribosome recycling. Nat. Struct. Mol. Biol. *14*, 727–732.

Boucher, H.W., Talbot, G.H., Bradley, J.S., Edwards, J.E., Gilbert, D., Rice, L.B., Scheld, M., Spellberg, B., and Bartlett, J. (2009). Bad bugs, no drugs: no ESKAPE! an update from the Infectious Diseases Society of America. Clin. Infect. Dis. *48*, 1–12.

Brodersen, D.E., Clemons, W.M., Jr., Carter, A.P., Morgan-Warren, R.J., Wimberly, B.T., and Ramakrishnan, V. (2000). The structural basis for the action of the antibiotics tetracycline, pactamycin, and hygromycin B on the 30S ribosomal subunit. Cell *103*, 1143–1154.

Bulkley, D., Johnson, F., and Steitz, T.A. (2012). The antibiotic thermorubin inhibits protein synthesis by binding to inter-subunit bridge B2a of the ribosome. J. Mol. Biol. *416*, 571–578.

Byrgazov, K., Vesper, O., and Moll, I. (2013). Ribosome heterogeneity: another level of complexity in bacterial translation regulation. Curr. Opin. Microbiol. *16*, 133–139.

Carter, A.P., Clemons, W.M., Brodersen, D.E., Morgan-Warren, R.J., Wimberly, B.T., and Ramakrishnan, V. (2000). Functional insights from the structure of the 30S ribosomal subunit and its interactions with antibiotics. Nature *407*, 340–348.

Chen, V.B., Arendall, W.B., 3rd, Headd, J.J., Keedy, D.A., Immormino, R.M., Kapral, G.J., Murray, L.W., Richardson, J.S., and Richardson, D.C. (2010). MolProbity: all-atom structure validation for macromolecular crystallography. Acta Crystallogr. D Biol. Crystallogr. *66*, 12–21.

Cocozaki, A.I., Altman, R.B., Huang, J., Buurman, E.T., Kazmirski, S.L., Doig, P., Prince, D.B., Blanchard, S.C., Cate, J.H., and Ferguson, A.D. (2016). Resistance mutations generate divergent antibiotic susceptibility profiles against translation inhibitors. Proc. Natl. Acad. Sci. U S A *113*, 8188–8193.

Cornish, P.V., Ermolenko, D.N., Noller, H.F., and Ha, T. (2008). Spontaneous intersubunit rotation in single ribosomes. Mol. Cell *30*, 578–588.

Demeshkina, N., Jenner, L., Westhof, E., Yusupov, M., and Yusupova, G. (2012). A new understanding of the decoding principle on the ribosome. Nature *484*, 256–259.

Dethlefsen, L., Huse, S., Sogin, M.L., and Relman, D.A. (2008). The pervasive effects of an antibiotic on the human gut microbiota, as revealed by deep 16S rRNA sequencing. PLoS Biol. *6*, e280.

Deuerling, E., Gamerdinger, M., and Kreft, S.G. (2019). Chaperone interactions at the ribosome. Cold Spring Harb. Perspect. Biol. *11*, a033977.

Dijkshoorn, L., Nemec, A., and Seifert, H. (2007). An increasing threat in hospitals: multidrug-resistant *Acinetobacter baumannii*. Nat. Rev. Microbiol. *5*, 939–951.

Emsley, P., and Cowtan, K. (2004). Coot: model-building tools for molecular graphics. Acta Crystallogr. D Biol. Crystallogr. *60*, 2126–2132.

Eyal, Z., Matzov, D., Krupkin, M., Wekselman, I., Paukner, S., Zimmerman, E., Rozenberg, H., Bashan, A., and Yonath, A. (2015). Structural insights into species-specific features of the ribosome from the pathogen *Staphylococcus aureus*. Proc. Natl. Acad. Sci. U S A *112*, E5805–E5814.

Falagas, M.E., and Bliziotis, I.A. (2007). Pandrug-resistant Gram-negative bacteria: the dawn of the post-antibiotic era? Int. J. Antimicrob. Agents *29*, 630–636.

Fischer, N., Neumann, P., Konevega, A.L., Bock, L.V., Ficner, R., Rodnina, M.V., and Stark, H. (2015). Structure of the *E. coli* ribosome-EF-Tu complex at <3 A resolution by Cs-corrected cryo-EM. Nature *520*, 567–570.

Fishbain, J., and Peleg, A.Y. (2010). Treatment of acinetobacter infections. Clin. Infect. Dis. *51*, 79–84.

Fournier, P.E., Vallenet, D., Barbe, V., Audic, S., Ogata, H., Poirel, L., Richet, H., Robert, C., Mangenot, S., Abergel, C., et al. (2006). Comparative genomics of multidrug resistance in *Acinetobacter baumannii*. PLoS Genet. *2*, e7.

Goddard, T.D., Huang, C.C., Meng, E.C., Pettersen, E.F., Couch, G.S., Morris, J.H., and Ferrin, T.E. (2018). UCSF ChimeraX: meeting modern challenges in visualization and analysis. Protein Sci. *27*, 14–25.

Halfon, Y., Jimenez-Fernandez, A., La Rosa, R., Espinosa Portero, R., Krogh Johansen, H., Matzov, D., Eyal, Z., Bashan, A., Zimmerman, E., Belousoff, M., et al. (2019a). Structure of *Pseudomonas aeruginosa* ribosomes from an aminoglycoside-resistant clinical isolate. Proc. Natl. Acad. Sci. U S A *116*, 22275–22281.

Halfon, Y., Matzov, D., Eyal, Z., Bashan, A., Zimmerman, E., Kjeldgaard, J., Ingmer, H., and Yonath, A. (2019b). Exit tunnel modulation as resistance mechanism of *S. aureus* erythromycin resistant mutant. Sci. Rep. *9*, 11460.

Hentschel, J., Burnside, C., Mignot, I., Leibundgut, M., Boehringer, D., and Ban, N. (2017). The complete structure of the *Mycobacterium smegmatis* 70S ribosome. Cell Rep. *20*, 149–160.

Howard, A., O'Donoghue, M., Feeney, A., and Sleator, R.D. (2012). *Acinetobacter baumannii*: an emerging opportunistic pathogen. Virulence *3*, 243–250.

James, N.R., Brown, A., Gordiyenko, Y., and Ramakrishnan, V. (2016). Translational termination without a stop codon. Science *354*, 1437–1440.

Jenner, L., Starosta, A.L., Terry, D.S., Mikolajka, A., Filonava, L., Yusupov, M., Blanchard, S.C., Wilson, D.N., and Yusupova, G. (2013). Structural basis for potent inhibitory activity of the antibiotic tigecycline during protein synthesis. Proc. Natl. Acad. Sci. U S A *110*, 3812–3816.

Jomaa, A., Boehringer, D., Leibundgut, M., and Ban, N. (2016). Structures of the *E. coli* translating ribosome with SRP and its receptor and with the translocon. Nat. Commun. *7*, 10471.

Jubb, H.C., Higueruelo, A.P., Ochoa-Montano, B., Pitt, W.R., Ascher, D.B., and Blundell, T.L. (2017). Arpeggio: a web server for calculating and visualising interatomic interactions in protein structures. J. Mol. Biol. *429*, 365–371.

Khusainov, I., Vicens, Q., Bochler, A., Grosse, F., Myasnikov, A., Menetret, J.F., Chicher, J., Marzi, S., Romby, P., Yusupova, G., et al. (2016). Structure

of the 70S ribosome from human pathogen *Staphylococcus aureus*. Nucleic Acids Res. *44*, 10491–10504.

Laskowski, R.A., and Swindells, M.B. (2011). LigPlot+: multiple ligand-protein interaction diagrams for drug discovery. J. Chem. Inf. Model. *51*, 2778–2786.

Li, W., Ward, F.R., McClure, K.F., Chang, S.T., Montabana, E., Liras, S., Dullea, R.G., and Cate, J.H.D. (2019). Structural basis for selective stalling of human ribosome nascent chain complexes by a drug-like molecule. Nat. Struct. Mol. Biol. *26*, 501–509.

Liu, Q., and Fredrick, K. (2016). Intersubunit bridges of the bacterial ribosome. J. Mol. Biol. *428*, 2146–2164.

Magnet, S., and Blanchard, J.S. (2005). Molecular insights into aminoglycoside action and resistance. Chem. Rev. *105*, 477–498.

Mohan, S., Donohue, J.P., and Noller, H.F. (2014). Molecular mechanics of 30S subunit head rotation. Proc. Natl. Acad. Sci. U S A *111*, 13325–13330.

Montefour, K., Frieden, J., Hurst, S., Helmich, C., Headley, D., Martin, M., and Boyle, D.A. (2008). *Acinetobacter baumannii*: an emerging multidrug-resistant pathogen in critical care. Crit. Care Nurse *28*, 15–25, quiz 26.

Morgan, C.E., Huang, W., Rudin, S.D., Taylor, D.J., Kirby, J.E., Bonomo, R.A., and Yu, E.W. (2020). Cryo-electron microscopy structure of the acinetobacter baumannii 70S ribosome and implications for new antibiotic development. mBio *11*, https://doi.org/10.1128/mBio.03117-19.

Nakane, T., Kimanius, D., Lindahl, E., and Scheres, S.H. (2018). Characterisation of molecular motions in cryo-EM single-particle data by multi-body refinement in RELION. eLife *7*, e36861.

Noeske, J., Wasserman, M.R., Terry, D.S., Altman, R.B., Blanchard, S.C., and Cate, J.H. (2015). High-resolution structure of the *Escherichia coli* ribosome. Nat. Struct. Mol. Biol. *22*, 336–341.

Ogle, J.M., Brodersen, D.E., Clemons, W.M., Jr., Tarry, M.J., Carter, A.P., and Ramakrishnan, V. (2001). Recognition of cognate transfer RNA by the 30S ribosomal subunit. Science *292*, 897–902.

Peleg, A.Y., Seifert, H., and Paterson, D.L. (2008). *Acinetobacter baumannii*: emergence of a successful pathogen. Clin. Microbiol. Rev. *21*, 538–582.

Perez, F., Endimiani, A., and Bonomo, R.A. (2008). Why are we afraid of *Acinetobacter baumannii*? Expert Rev. Anti. Infect. Ther. *6*, 269–271.

Perez, F., Hujer, A.M., Hujer, K.M., Decker, B.K., Rather, P.N., and Bonomo, R.A. (2007). Global challenge of multidrug-resistant *Acinetobacter baumannii*. Antimicrob. Agents Chemother. *51*, 3471–3484.

Pettersen, E.F., Goddard, T.D., Huang, C.C., Couch, G.S., Greenblatt, D.M., Meng, E.C., and Ferrin, T.E. (2004). UCSF Chimera–a visualization system for exploratory research and analysis. J. Comput. Chem. *25*, 1605–1612.

Pioletti, M., Schlunzen, F., Harms, J., Zarivach, R., Gluhmann, M., Avila, H., Bashan, A., Bartels, H., Auerbach, T., Jacobi, C., et al. (2001). Crystal structures of complexes of the small ribosomal subunit with tetracycline, edeine and IF3. EMBO J. *20*, 1829–1839.

Poirel, L., and Nordmann, P. (2006). Carbapenem resistance in *Acinetobacter baumannii*: mechanisms and epidemiology. Clin. Microbiol. Infect. *12*, 826–836.

Polikanov, Y.S., Melnikov, S.V., Soll, D., and Steitz, T.A. (2015). Structural insights into the role of rRNA modifications in protein synthesis and ribosome assembly. Nat. Struct. Mol. Biol. *22*, 342–344.

Potron, A., Poirel, L., and Nordmann, P. (2015). Emerging broad-spectrum resistance in *Pseudomonas aeruginosa* and *Acinetobacter baumannii*: mechanisms and epidemiology. Int. J. Antimicrob. Agents *45*, 568–585.

Ratje, A.H., Loerke, J., Mikolajka, A., Brunner, M., Hildebrand, P.W., Starosta, A.L., Donhofer, A., Connell, S.R., Fucini, P., Mielke, T., et al. (2010). Head swivel on the ribosome facilitates translocation by means of intra-subunit tRNA hybrid sites. Nature *468*, 713–716.

Rozov, A., Demeshkina, N., Khusainov, I., Westhof, E., Yusupov, M., and Yusupova, G. (2016). Novel base-pairing interactions at the tRNA wobble position crucial for accurate reading of the genetic code. Nat. Commun. *7*, 10457.

Schedlbauer, A., Kaminishi, T., Ochoa-Lizarralde, B., Dhimole, N., Zhou, S., Lopez-Alonso, J.P., Connell, S.R., and Fucini, P. (2015). Structural characterization of an alternative mode of tigecycline binding to the bacterial ribosome. Antimicrob. Agents Chemother. *59*, 2849–2854.

Schluenzen, F., Tocilj, A., Zarivach, R., Harms, J., Gluehmann, M., Janell, D., Bashan, A., Bartels, H., Agmon, I., Franceschi, F., et al. (2000). Structure of functionally activated small ribosomal subunit at 3.3 angstroms resolution. Cell *102*, 615–623.

Schlunzen, F., Zarivach, R., Harms, J., Bashan, A., Tocilj, A., Albrecht, R., Yonath, A., and Franceschi, F. (2001). Structural basis for the interaction of antibiotics with the peptidyl transferase centre in eubacteria. Nature *413*, 814–821.

Schneider, C.A., Rasband, W.S., and Eliceiri, K.W. (2012). NIH Image to ImageJ: 25 years of image analysis. Nat. Methods *9*, 671–675.

Schuwirth, B.S., Day, J.M., Hau, C.W., Janssen, G.R., Dahlberg, A.E., Cate, J.H., and Vila-Sanjurjo, A. (2006). Structural analysis of kasugamycin inhibition of translation. Nat. Struct. Mol. Biol. *13*, 879–886.

Schwede, T., Kopp, J., Guex, N., and Peitsch, M.C. (2003). SWISS-MODEL: an automated protein homology-modeling server. Nucleic Acids Res. *31*, 3381–3385.

Sohmen, D., Chiba, S., Shimokawa-Chiba, N., Innis, C.A., Berninghausen, O., Beckmann, R., Ito, K., and Wilson, D.N. (2015). Structure of the *Bacillus subtilis* 70S ribosome reveals the basis for species-specific stalling. Nat. Commun. *6*, 6941.

Tacconelli, E., Carrara, E., Savoldi, A., Harbarth, S., Mendelson, M., Monnet, D.L., Pulcini, C., Kahlmeter, G., Kluytmans, J., Carmeli, Y., et al. (2018). Discovery, research, and development of new antibiotics: the WHO priority list of antibiotic-resistant bacteria and tuberculosis. Lancet Infect. Dis. *18*, 318–327.

Thompson, R.F., Iadanza, M.G., Hesketh, E.L., Rawson, S., and Ranson, N.A. (2018). Collection, pre-processing and on-the-fly analysis of data for high-resolution, single-particle cryo-electron microscopy. Nat. Protoc. *14*, 100–118.

Tsai, A., Uemura, S., Johansson, M., Puglisi, E.V., Marshall, R.A., Aitken, C.E., Korlach, J., Ehrenberg, M., and Puglisi, J.D. (2013). The impact of aminoglycosides on the dynamics of translation elongation. Cell Rep. *3*, 497–508.

Wang, L., Pulk, A., Wasserman, M.R., Feldman, M.B., Altman, R.B., Cate, J.H., and Blanchard, S.C. (2012). Allosteric control of the ribosome by small-molecule antibiotics. Nat. Struct. Mol. Biol. *19*, 957–963.

Wilson, D.N. (2009). The A-Z of bacterial translation inhibitors. Crit. Rev. Biochem. Mol. Biol. *44*, 393–433.

Wilson, D.N. (2014). Ribosome-targeting antibiotics and mechanisms of bacterial resistance. Nat. Rev. Microbiol. *12*, 35–48.

Wimberly, B.T., Brodersen, D.E., Clemons, W.M., Jr., Morgan-Warren, R.J., Carter, A.P., Vonrhein, C., Hartsch, T., and Ramakrishnan, V. (2000). Structure of the 30S ribosomal subunit. Nature *407*, 327–339.

Wright, H., Bonomo, R.A., and Paterson, D.L. (2017). New agents for the treatment of infections with Gram-negative bacteria: restoring the miracle or false dawn? Clin. Microbiol. Infect. *23*, 704–712.

Yang, K., Chang, J.Y., Cui, Z., Li, X., Meng, R., Duan, L., Thongchol, J., Jakana, J., Huwe, C.M., Sacchettini, J.C., et al. (2017). Structural insights into species-specific features of the ribosome from the human pathogen *Mycobacterium tuberculosis*. Nucleic Acids Res. *45*, 10884–10894.

Zhang, K. (2016). Gctf: real-time CTF determination and correction. J. Struct. Biol. *193*, 1–12.

Zheng, S.Q., Palovcak, E., Armache, J.P., Verba, K.A., Cheng, Y., and Agard, D.A. (2017). MotionCor2: anisotropic correction of beam-induced motion for improved cryo-electron microscopy. Nat. Methods *14*, 331–332.

Zivanov, J., Nakane, T., Forsberg, B.O., Kimanius, D., Hagen, W.J., Lindahl, E., and Scheres, S.H. (2018). New tools for automated high-resolution cryo-EM structure determination in RELION-3. eLife *7*, e42166.

STAR★METHODS

KEY RESOURCES TABLE

REAGENT or RESOURCE	SOURCE	IDENTIFIER
Bacterial and Virus Strains		
Acinetobacter baumannii	ATCC	ATCC 19606
Chemicals, Peptides, and Recombinant Proteins		
amikacin	Cayman chemical	Cat# 15405
tigecycline	LKT Labs	Cat# T3324
Deposited Data		
Motion corrected micrographs of amikacin-ribosome	This study	EMPIAR-10406
Motion corrected micrographs of tigecycline-ribosome	This study	EMPIAR-10407
Density map of amikacin-ribosome 50S	This study	EMD-10809
Density map of amikacin-ribosome 30S body	This study	EMD-10869
Density map of amikacin-ribosome 30S head	This study	EMD-10892
Density map of tigecycline-ribosome 50S	This study	EMD-10898
Density map of tigecycline-ribosome 30S body	This study	EMD-10914
Density map of tigecycline-ribosome 30S head	This study	EMD-10915
Coordinates of amikacin-ribosome 50S	This study	PDB 6YHS
Coordinates of amikacin-ribosome 30S body	This study	PDB 6YPU
Coordinates of amikacin-ribosome 30S head	This study	PDB 6YS5
Coordinates of tigecycline-ribosome 50S	This study	PDB 6YSI
Coordinates of tigecycline-ribosome 30S body	This study	PDB 6YT9
Coordinates of tigecycline-ribosome 30S head	This study	PDB 6YTF
Homology model template for initial model building. *E. coli* 70S ribosome	(James et al., 2016)	Protein Data Bank 5MDZ
fMet-tRNA atomic model for modelling of E-site tRNA	(Fischer et al., 2015)	Protein Data Bank 5AFI
Software and Algorithms		
RELION 3.0	(Zivanov et al., 2018)	https://www3.mrc-lmb.cam.ac.uk/relion/index.php?title=Main_Page
MotionCor2	(Zheng et al., 2017)	https://emcore.ucsf.edu/ucsf-motioncor2
gCTF v1.18	(Zhang, 2016)	https://www.mrc-lmb.cam.ac.uk/kzhang/Gctf/
SWISS model	(Schwede et al., 2003)	https://swissmodel.expasy.org/
Coot 0.8.9.2	(Emsley and Cowtan, 2004)	https://www2.mrc-lmb.cam.ac.uk/personal/pemsley/coot/
Phenix v1.17.1-3660	(Adams et al., 2010)	https://www.phenix-online.org/
MolProbity	(Chen et al., 2010)	http://molprobity.biochem.duke.edu/
UCSF ChimeraX-0.9	(Goddard et al., 2018)	https://www.cgl.ucsf.edu/chimerax/
ImageJ	(Schneider et al., 2012)	https://imagej.nih.gov/ij/
Pymol 2.3.2		https://pymol.org/2/

(*Continued on next page*)

Continued

REAGENT or RESOURCE	SOURCE	IDENTIFIER
MolView		http://molview.org/
Arpeggio	(Jubb et al., 2017)	http://biosig.unimelb.edu.au/arpeggioweb/
LigPlot+ v2.1	(Laskowski and Swindells, 2011)	https://www.ebi.ac.uk/thornton-srv/software/LigPlus/

RESOURCE AVAILABILITY

Lead Contact

Further information and requests for resources and reagents should be directed to and will be fulfilled by the Lead Contact, Neil Ranson (n.a.ranson@leeds.ac.uk).

Materials Availability

The study did not generate new unique reagents.

Data and Code Availability

CryoEM motion-corrected micrographs generated in this study are available in EMPIAR, along with extracted particle stacks (EMPIAR-10406 and 10407 for amikacin and tigecycline respectively). CryoEM multibody refinement maps are available in the EMDB for amikacin (EMD-10809 (50S), EMD-10869 (30S body) and EMD-10892 (30S head)) and for tigecycline (EMD-10898 (50S), EMD-10914 (30S body) and EMD-10915 (30S head)). Corresponding atomic models are available in the PDB for amikacin (6YHS (50S), 6YPU (30S body), and 6YS5 (30S head)) and for tigecycline (6YSI (50S), 6YT9 (30S body), and 6YTF (30S head)). Half-maps, masks used for multibody refinement and post-processing, and pre-multibody consensus reconstructions are all available as part of the EMDB entries. See Key Resources Table for more details.

EXPERIMENTAL MODEL AND SUBJECT DETAILS

A. baumannii strain 19606 was used for these studies, and cells were grown in LB media in shaking incubators. Additional details are provided in the Method Details section.

METHOD DETAILS

70S Ribosome Purification

Two litres of *A. baumannii* type strain ATCC 19606 were grown at 37ºC in LB media and harvested at early-mid log phase (OD_{600} of ~0.5). The cell pellet was washed with 10 mM Tris-HCl pH 7.5 and stored at -80°C. Each 1 g of cell pellet was resuspended in 2 mL lysis buffer (20 mM HEPES-KOH pH 7.5, 100 mM NH_4Cl, 20 mM $Mg(OAc)_2$, 0.5 mM EDTA, 1 mM DTT) supplemented with cOmplete protease inhibitor cocktail (Roche, one tablet per 10 mL) and RNase-free DNase (300 U). The resuspension was lysed using a cell disruptor (two passes at 25K psi) and cleared by centrifugation at 30,000 x *g* for 30 minutes. The top 80% of the supernatant was collected and recentrifuged at 30,000 x *g* for 30 minutes, and the resulting supernatant layered onto a sucrose cushion buffer (10 mM HEPES-KOH pH 7.5, 500 mM KCl, 25 mM $Mg(OAc)_2$, 1.1 M sucrose (40% w/v), 0.5 mM EDTA, 1 mM DTT) and spun by ultracentifugation at 150,000 x *g* for 16 hours. The resulting pellet was gently resuspended in 200 μL of sucrose gradient buffer (10 mM HEPES-KOH pH 7.5, 100 mM KCl, 10 mM $Mg(OAc)_2$, 0.5 mM EDTA, 1 mM DTT) and layered on top of a 10-40% w/v sucrose density gradient (made by dissolving different amounts of sucrose in sucrose gradient buffer). Ultracentrifugation was subsequently carried out at 50,000 x *g* for 16 hours, and the fractions corresponding to the largest A_{260} peak were collected and dialysed into storage buffer (10 mM HEPES-KOH pH 7.5, 50 mM KCl, 10 mM NH_4Cl, 10 mM $Mg(OAc)_2$, 1 mM DTT) using a 20K molecular weight cutoff Slide-A-Lyzer MINI Dialysis Device (Thermo Scientific), flash frozen in liquid nitrogen, and stored at −80°C.

CryoEM

Purified 70S ribosomes (120 nM) were incubated with amikacin (100 μM) or purified 70S ribosomes (240 nM) were incubated with tigecycline (71.7 μM) at room temperature for 30 minutes. Quantifoil grids (R1.2/1.3, 400 mesh, with a 2 nm carbon layer) were glow discharged (10 mA, 30s, Quorum GloQube), 3 μL of the drug-ribosome reaction mixture applied, excess sample immediately blotted off and vitrification performed by plunging into liquid nitrogen-cooled liquid ethane at 100% humidity and 4°C using an FEI Vitrobot Mark IV (ThermoFisher). Data for both samples were collected on a ThermoFisher Titan Krios electron microscope (Astbury Biostructure Laboratory, University of Leeds) at 300 kV. Data collection was set up as described previously (Thompson et al., 2018). For the amikacin-ribosome sample, an electron dose of 58 $e^-/Å^2$ was applied, split into 1.16 $e^-/Å^2$ dose per frame across a 10 s exposure recorded by a Gatan K2 summit detector in counting mode with an object sampling of 1.07 Å/pixel. A magnification of 130,000

across a defocus range of -0.8 to -2.7 μm was used. For the tigecycline-ribosome sample, an electron dose of 62 $e^-/Å^2$ was applied, split into 1.44 $e^-/Å^2$ dose per frame across a 1.1 s exposure recorded by a ThermoFisher Falcon 3EC detector in integrating mode with an object sampling of 1.065 Å/pixel. A magnification of 75,000 across a defocus range of -0.8 to -2.6 μm was used. 2717 and 6228 micrograph movies of the amikacin- and tigecycline-ribosome samples were collected respectively, and following culling of micrographs with poor ice quality, 554 and 6228 micrograph movies remained respectively (Table S1).

Image Processing

Drift-corrected and dose-corrected averages of each movie were created using MOTIONCOR2 (Zheng et al., 2017) and the contrast transfer functions determined using Gctf (Zhang, 2016). All subsequent image processing steps were carried out using RELION 3.0 (Zivanov et al., 2018). Particles were picked using Laplacian-of-Gaussian autopicking and reference-free 2D classification and 3D classification performed on binned-by-4 particles to remove junk images. The remaining particles were re-extracted without binning and aligned and refined in 3D using a 60 Å low-passed filtered *ab initio* starting model made by a stochastic gradient descent procedure. The number of particles feeding into the final reconstructions were 51,958 and 231,159 for the amikacin-ribosome and tigecycline-ribosome samples respectively. Multibody refinement was performed using soft extended masks to define the 50S, 30S body and 30S head as rigid bodies (Figure S2). This procedure uses iteratively improved partial signal subtraction and focussed refinement to generate higher quality reconstructions for each body (Nakane et al., 2018). The resulting reconstructions were subjected to post-processing to mask out solvent and estimate and correct for the B-factors. The final resolutions were estimated using the gold-standard Fourier shell correlation (FSC = 0.143) criterion. Local resolution was estimated using RELION 3.0 (Figures S1 and S2).

Atomic Model Building and Refinement

The cryoEM structure of an *E. coli* ribosome (PDB 5MDZ) (James et al., 2016) was used as a starting reference for modelling the *A. baumannii* 23S, 16S and 5S rRNAs into the post-processed multibody reconstructions. Homology models were generated for the ribosomal proteins using the SWISS model server (Schwede et al., 2003) and rigid-body fit into the reconstructions in UCSF Chimera (Pettersen et al., 2004) using PDB 5MDZ to guide placement. The models were inspected using COOT (Emsley and Cowtan, 2004), and in all three amikacin-ribosome multibody reconstructions and the tigecycline-ribosome 50S and 30S body multibody reconstructions, regions of protein where side chains could not be resolved were modelled without side chains, and regions where the protein or rRNA backbone could not be traced were deleted. The tigecycline-ribosome 30S head reconstruction was of slightly poorer quality than the other maps and so such highly stringent trimming of the model was not carried out. Instead, the full amikacin-ribosome 30S head model was predicted to be a good approximation for the tigecycline-ribosome 30S head and hence was used as a starting model and retained with no further deletion of backbone or side chains. Density for tRNA was present in the ribosome E-site, likely corresponding to a mixture of different tRNAs, was modelled using fMet-tRNA from *E. coli* (PDB 5AFI) (Fischer et al., 2015) as a starting model. Only the regions near the 50S and 30S subunits which had resolved nucleotide density were retained. Density corresponding to a short mRNA at the E-site was also resolved, and this was modelled as a short polyuridine chain (Figure S5). COOT was used to manually adjust the models to improve map and rotamer fit and reduce Ramachandran outliers, before iterative rounds of model refinement and manual model editing were carried out using PHENIX real space refine (Adams et al., 2010) and COOT respectively. Models were validated using MolProbity (Chen et al., 2010) within PHENIX and PDB OneDep (Berman et al., 2003). Throughout the process, the models for the 50S, 30S body and 30S head were kept separate and refined independently into their corresponding maps, as this reflects the data from the multibody refinement procedure which generates independent reconstructions (Nakane et al., 2018). Details of the final model are found in Table S2. Model refinement and validation statistics are found in Table 1.

Figures and Model Analysis

Figures were made using UCSF ChimeraX (Goddard et al., 2018), ImageJ (Schneider et al., 2012), , LigPlot+ (Laskowski and Swindells, 2011), PyMol, and MolView. Interactions between the tigecycline molecules in the secondary binding site and the surrounding ribosome were calculated and visualised using Arpeggio (Jubb et al., 2017).

QUANTIFICATION AND STATISTICAL ANALYSIS

All cryoEM data sets were processed using RELION (Table S1). All resolutions reported are based on the "gold-standard" FSC 0.143 criterion (Figure S1 and S2). FSC curves were calculated using soft-edged masks. Refinement statistics of all atomic models are summarized in Table S1. These models were also evaluated based on MolProbity scores (Chen et al (2010)) and Ramachandran plots.

Structure, Volume *28*

Supplemental Information

Structure of the 70S Ribosome from the Human Pathogen *Acinetobacter baumannii* in Complex with Clinically Relevant Antibiotics

David Nicholson, Thomas A. Edwards, Alex J. O'Neill, and Neil A. Ranson

Table S1. **CryoEM data collection statistics, related to Figures 1-5.**

	**amikacin-ribosome**	**tigecycline-ribosome**
microscope	Titan Krios	Titan Krios
camera	K2 Summit	Falcon 3EC
collection mode	counting	linear
voltage (kV)	300	300
nominal magnification	130,000	75,000
pixel size (Å/px)	1.07	1.065
defocus range (µm)	(-0.8)–(-2.7)	(-0.8)–(-2.6)
exposure time (s)	10	1.1
total dose (e^-/Å^2)	58	61.78
dose per frame (e^-/Å^2)	1.16	1.44
micrographs collected	2717	6228
micrographs with good ice	554	6228
number of particles	51958	231159
symmetry imposed	C1	C1
final resolution (Å)	2.8	2.6

Table S2. **Atomic model details. Related to Figure 1.**

**rRNA chains**					
**chain ID**	**rRNA name**	**GenBank accession number**	**locus tag**	**modelled residues**	**corresponding EM map**
1	23S	KL810966	DJ41_1033	2-868; 891-1034; 1101-1511; 1535-1712; 1726-2086; 2181-2891	50S
2	16S 5'	KL810966	DJ41_1036	3-77; 90-199; 210-837; 843-923	30S body
3	16S central	KL810966	DJ41_1036	924-1022; 1031-1384	30S head
4	16S 3'	KL810966	DJ41_1036	1385-1530	30S body
5	5S	KL810966	DJ41_1032	2-116	50S

**protein chains**						
**chain ID**	**protein name**	**UniProt accession number**	**full size (residues)**	**modelled residues**	**corresponding EM map**	**notes**
A	uL2	D0CD00	274	2-273	50S	
B	uL3	D0CCZ7	212	2-212	50S	
C	uL4	D0CCZ8	200	2-200 (2-12 no side chains)	50S	
D	uL5	D0CD09	178	(2-177 no side chains)	50S	
E	uL6	D0CD12	177	2-176	50S	
F	uL13	D0CG35	142	1-142	50S	
G	uL14	D0CD07	122	1-122	50S	
H	uL15	D0CD16	146	2-145	50S	
I	uL16	D0CD04	137	1-137	50S	
J	bL17	D0CD23	125	1-119	50S	
K	uL18	D0CD13	116	2-116	50S	
L	bL19	D0CCR8	122	2-118	50S	
M	bL20	D0CA76	119	2-118	50S	
N	bL21	D0CDQ6	103	1-103	50S	
O	uL22	D0CD02	110	2-110	50S	
P	uL23	D0CCZ9	106	1-92	50S	
Q	uL24	D0CD08	105	2-103	50S	
R	bL25	D0C9L7	98	3-98	50S	
S	bL27	D0CDQ7	85	9-84	50S	
T	bL28	D0CAL0	78	2-78	50S	
U	uL29	D0CD05	65	2-62	50S	

V	uL30	D0CD15	58	1-57	50S	
W	bL31	D0CBZ8	74	(1-43 no side chains)	50S	tigecycline model only
X	bL32	D0C9K5	61	2-55	50S	
Y	bL33	D0CAL1	51	1-51	50S	
Z	bL34	D0CG06	44	1-44	50S	
a	bL35	D0CA77	64	2-64	50S	
b	bL36	D0CD18	38	1-38	50S	
c	uS2	D0CC74	250	(8-226 no side chains)	30S body	
d	uS3	D0CD03	250	2-211	30S head	
e	uS4	D0CD21	208	2-208 (23-30; 43-51 no side chains)	30S body	
f	uS5	D0CD14	165	10-164	30S body	
g	bS6	D0C5Z0	127	1-103	30S body	
h	uS7	D0C9P7	156	3-70; 97-145 (126-145 no side chains)	30S head	
i	uS8	D0CD11	131	2-131	30S body	
j	uS9	D0CG36	128	2-128	30S head	
k	uS10	D0CCZ6	103	4-103	30S head	
l	uS11	D0CD20	128	15-128	30S body	
m	uS12	D0C9P6	124	2-123	30S body	
n	uS13	D0CD19	118	2-116	30S head	
o	uS14	D0CD10	101	2-101	30S head	
p	uS15	D0CAU9	89	2-89	30S body	
q	bS16	D0CCR5	83	1-80	30S body	
r	uS17	D0CD06	85	5-83	30S body	
s	bS18	D0C5Y9	75	21-73	30S body	
t	uS19	D0CD01	91	2-84	30S head	
u	bS20	D0C7N1	88	2-87	30S body	
v	bS21	D0C5Q3	71	2-61 (2-36 no side chains)	30S body	

other chains			
chain ID	name	corresponding EM map	notes
6	tRNA 5'	50S	residues 2-6 from PDB 5AFI E-site fMet-tRNA, probably actually a mixture of tRNAs
7	tRNA central	30S head	residues 26-45 from PDB 5AFI E-site fMet-tRNA, probably actually a mixture of tRNAs
8	tRNA 3'	50S	residues 69-76 from PDB 5AFI E-site fMet-tRNA, probably actually a mixture of tRNAs
9	mRNA	30S head	4 nucleotide polyU mRNA model, probably actually a mixture of mRNAs

unmodelled proteins
uL1
bL9
uL10
uL11
bL12
bS1

Table S3. **Unique structural features of the *A. baumannii* ribosome. Related to Figure 2.**

chain	*A. baumannii* residue number	*E. coli* residue number	rRNA helix	details	scale of difference	quality of density
**rRNAs**						
23S	60-64	60-64	H6	different fold	minor	good
	99-105	99-105	H7	different fold	major	good
	135-147	135-147	H9	different fold	minor	fair
	263-284	263-289	H18	deletion	major	fair
	349-365	354-373	H18	deletion	major	good
	634-639	643-648	H31	different fold	minor	good
	921-925	930-935	H38	deletion	minor	good
	1130-1134	1140-1143	H41-42	insertion	minor	good
	1490-1503	1502-1514	H58	insertion	minor	fair
	1701-1738	1710-1749	H63	deletion	major	positions 1713-1725 not resolved
16S	70-98	68-102	h6	deletion	major	positions 78-89 not resolved
	153-165	157-169	h8	different fold	minor	fair
	175-189	179-193	h9	different fold	major	good
	447-479	451-482	h17	insertion	minor	fair
	1065-1081	1068-1084	h35-36	different fold	minor	good
	1252-1256	1255-1259	h41	different fold	minor	good
	1436-1440	1439-1443	h44	different fold	minor	fair
5S	5'- and 3'-ends	5'- and 3'-ends		shorter	minor	fair
	82-91	83-93	helix IV	deletion	major	good
**proteins**						
uL3	85-96	84-93		insertion	major	good
	104-109	101-106		different fold	major	good
uL6	15-23	15-23		different fold	minor	fair
	54-60	54-60		different fold	major	fair
uL14	88-92	88-93		deletion	minor	good
uL16	20-24	20-23		insertion	minor	good
uL18	54-64	53-65		deletion	major	fair
bL19	N-terminus	N-terminus		extension	major	good
uL22	C-terminus	C-terminus		deletion	minor	good
uL23	N-terminus	N-terminus		deletion	minor	fair
	65-74	66-75		different fold	minor	good
uL24	N-terminus	N-terminus		deletion	minor	good
	27-31	28-33		deletion	minor	good
	C-terminus	C-terminus		extension	minor	fair
bL25	11-16	9-12		insertion	minor	good
	83-90	79-86		different fold	minor	good
bL32	23-33	23-34		deletion	major	good
	51-55	52-56		different fold	minor	good
bL33	N-terminus	N-terminus		deletion	major	good
bL34	C-terminus	C-terminus		deletion	major	good
bL35	17-21	17-22		deletion	minor	good
uS3	59-65	59-65		different fold	minor	good
uS4	31-37	31-36		insertion	major	fair
	82-88	80-86		different fold	minor	good

uS8	53-57	53-56		insertion	minor	good
uS15	N-terminus	N-terminus		different fold	minor	good
bS16	44-50	44-50		different fold	minor	good
uS17	N-terminus	N-terminus		different fold	minor	good

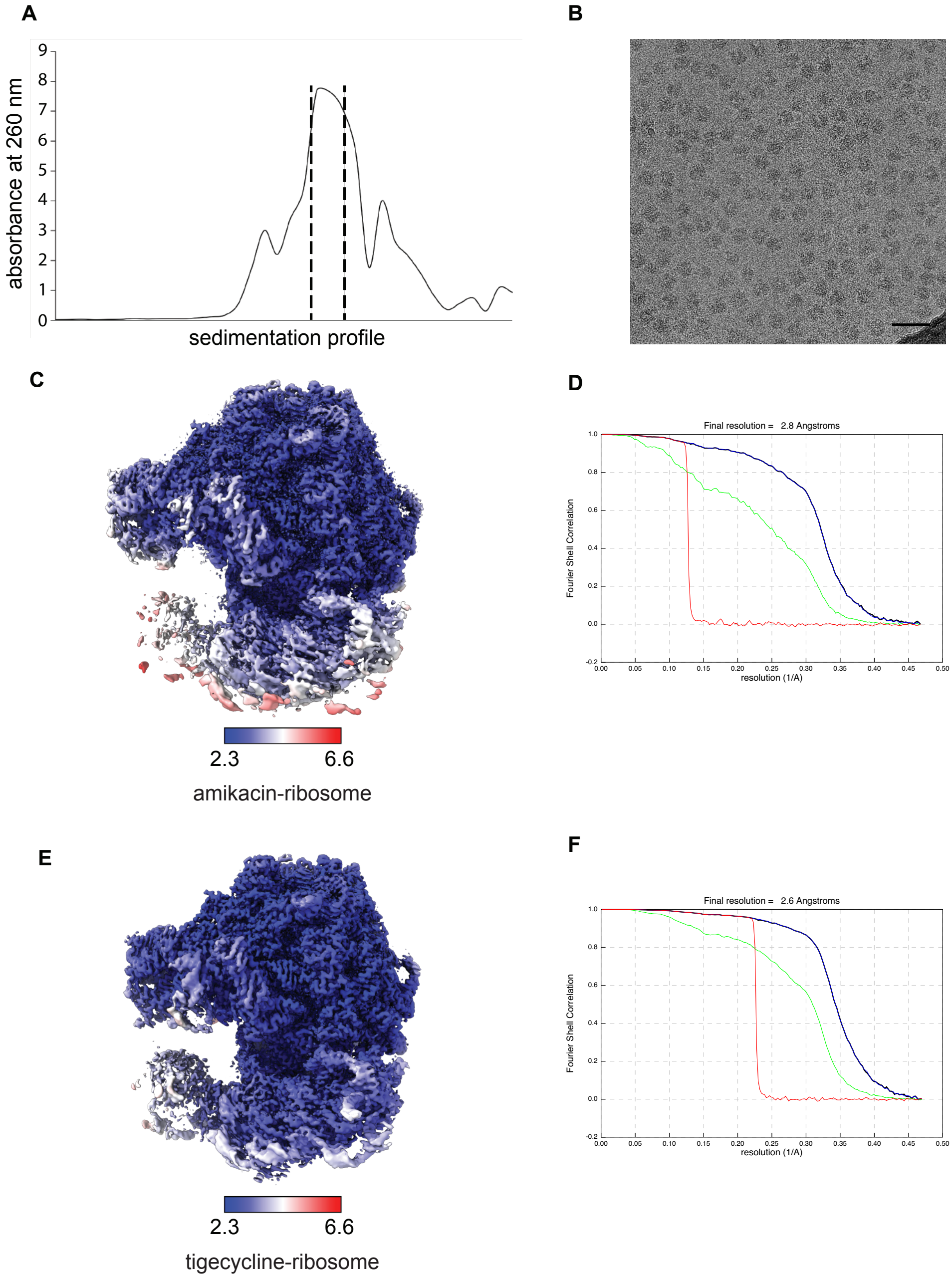

Figure S1. **Sample characterisation and cryoEM image processing, related to Figures 1-5.** A) Sucrose gradient A_{260} sedimentation profile, showing the peak where fractions were collected. B) Representative cryoEM micrograph of the *A. baumannii* ribosome-amikacin sample, scale bar 50 nm. C) CryoEM reconstruction of the amikacin-ribosome structure, filtered by local resolution. D) FSC curves as a function of resolution for the amikacin-ribosome struc-ture. The resolution that corresponds to an FSC coefficient of 0.143 is 2.8 Å. E) CryoEM reconstruction of the *A. baumannii* ribosome-tigecycline structure, filtered by local resolution. F) FSC curves as a function of resolution for the tigecycline-ribosome structure. The resolution that corresponds to an FSC coefficient of 0.143 is 2.6 Å. Maps coloured by local resolution, from 2.3 Å (blue) to 6.6 Å (red). FSC curves are shown for phase-randomised maps (red), unmasked maps (green), masked maps (blue), and masked maps after correction for mask convolution effects (black).

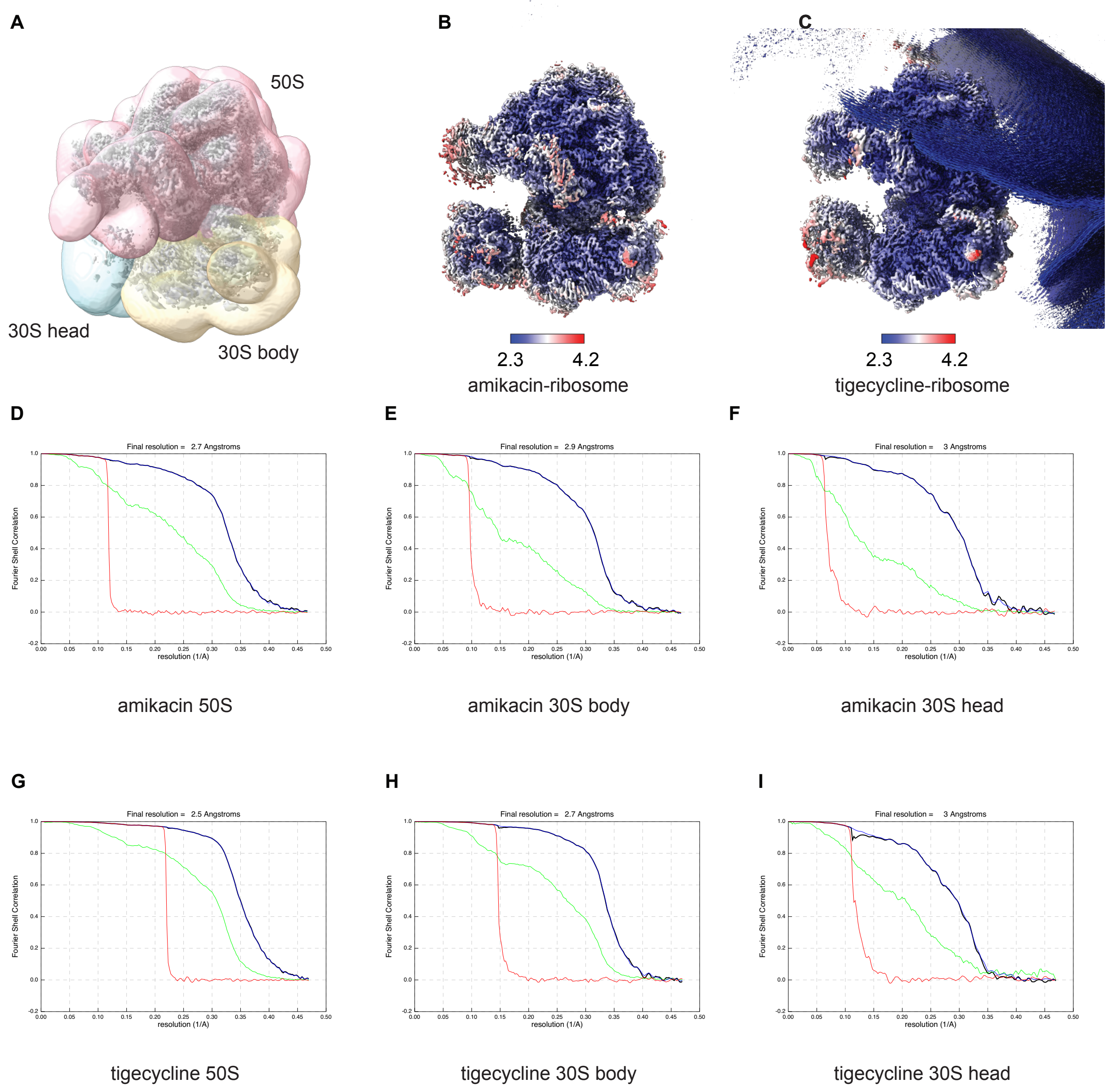

Figure S2. **CryoEM multibody refinement, related to Figure 1-5.** A) Masks used to specify the 50S (pink), 30S body (brown) and 30S head (blue) rigid bodies, shown around the pre-multibody consensus reconstruction of the *A. baumannii* ribosome-amikacin complex (grey). B) CryoEM reconstructions of the three bodies comprising the amikacin-ri-bosome structure after multibody refinement, filtered by local resolution. C) CryoEM reconstructions of the three bodies comprising the tigecycline-ribosome structure after multibody refinement, filtered by local resolution. Maps coloured by local resolution, from 2.3 Å (blue) to 4.2 Å (red). Note that padding in Fourier space was not performed in order to save memory, resulting in artefacts around the edge of the box. This noise was masked out before undertaking model building and refinement. D-F) FSC curves as a function of resolution for the amika-cin-ribosome multibody structures. G-I) FSC curves as a function of resolution for the tigecycline-ribosome multi-body structures. FSC curves are shown for phase-randomised maps (red), unmasked maps (green), masked maps (blue), and masked maps after correction for mask convolution effects (black).

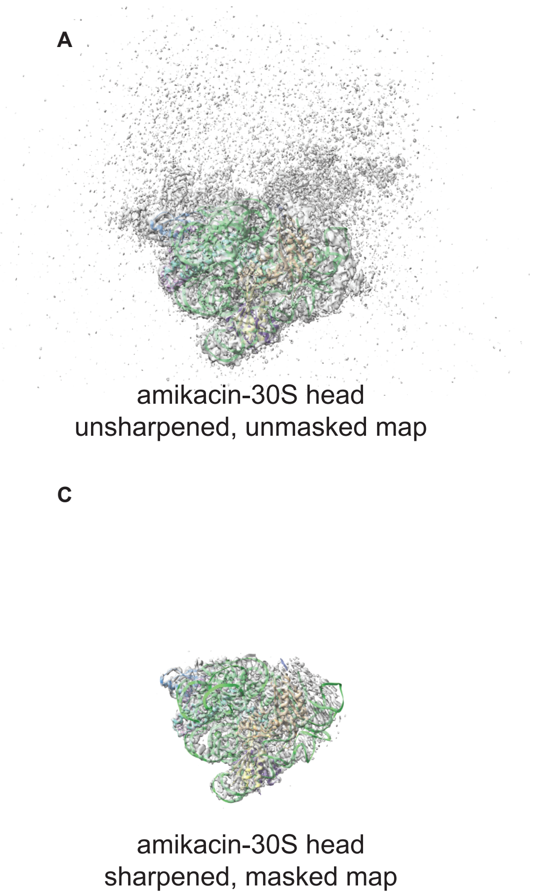

Figure S3. **Comparison of the 30S head EM density of the *A. baumannii* ribosome-amikacin and *A. baumannii* ribosome-tigecycline structures, related to Figure 1-5.** A) Atomic model and EM density of the 30S head of the amika-cin-ribosome complex. B) Atomic model and EM density of the 30S head of the tigecycline-ribosome complex. Semi-transparent grey densities correspond to the unsharpened and unmasked outputs of multibody refinement. C) Atomic model and EM of the 30S head of the amikacin-ribosome complex. D) Atomic model and EM density of the 30S head of the tigecycline-ribosome complex. Semi-transparent grey densities correspond to the sharp-ened and masked maps after post-processing of the multibody refinement outputs.

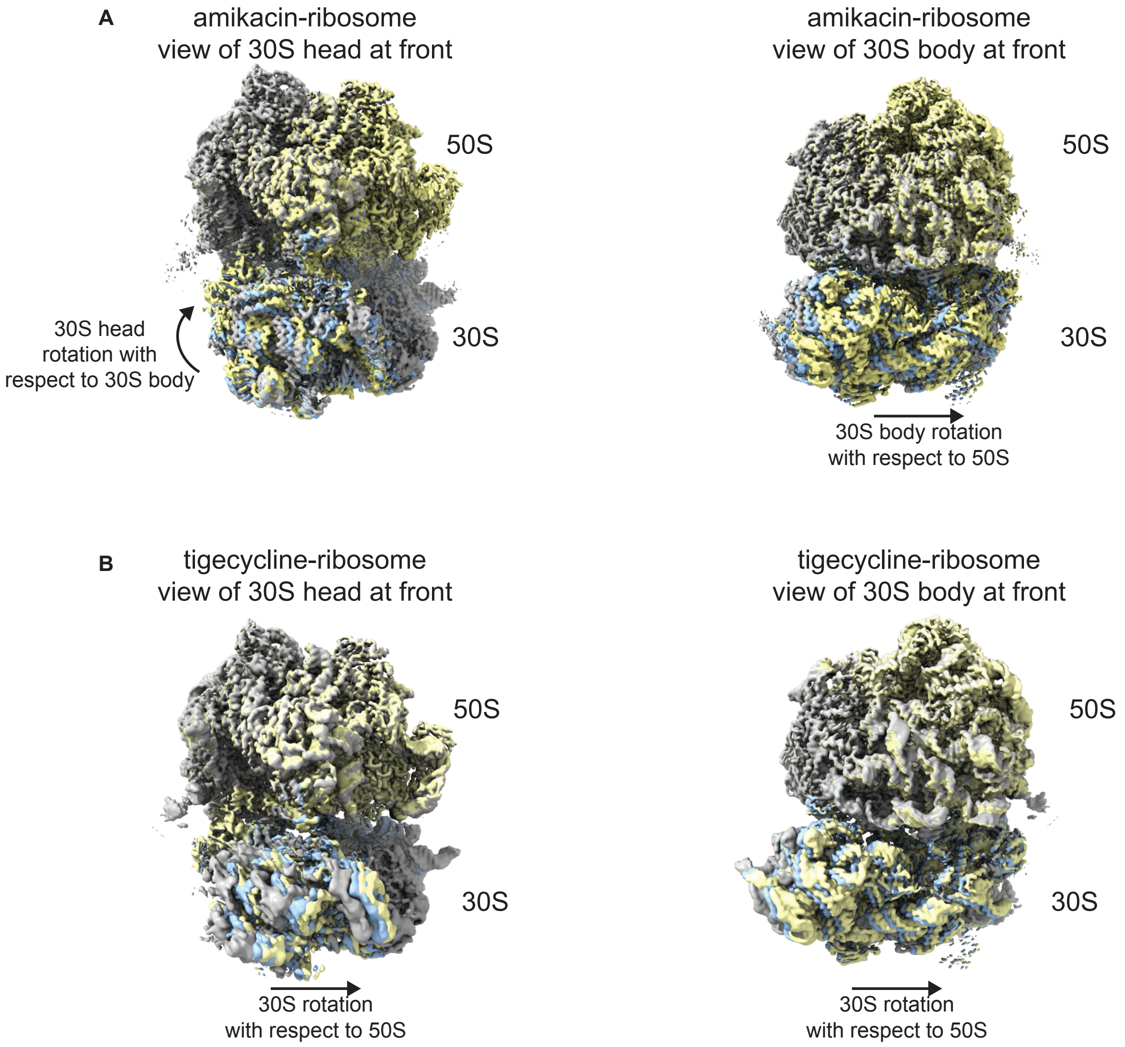

Figure S4. **Comparison of the components contributing the largest variations to the data for the *A. baumannii* ribosome-amikacin and ribosome-tigecycline complexes, related to Figure 1-5.** A) The principle component describ-ing 27% of the variation in the data for the amikacin-ribosome complex. Movement is described along a trajecto-ry from grey to blue to yellow EM density. This component describes a rotation of the 30S head as well as inter-subunit rotation between the 50S and 30S body. B) The principle component describing 28% of the varia-tion in the data for the tigecycline-ribosome complex. Movement is described along a trajectory from grey to blue to yellow EM density. This component describes predominantly inter-subunit rotation between the 50S and 30S.

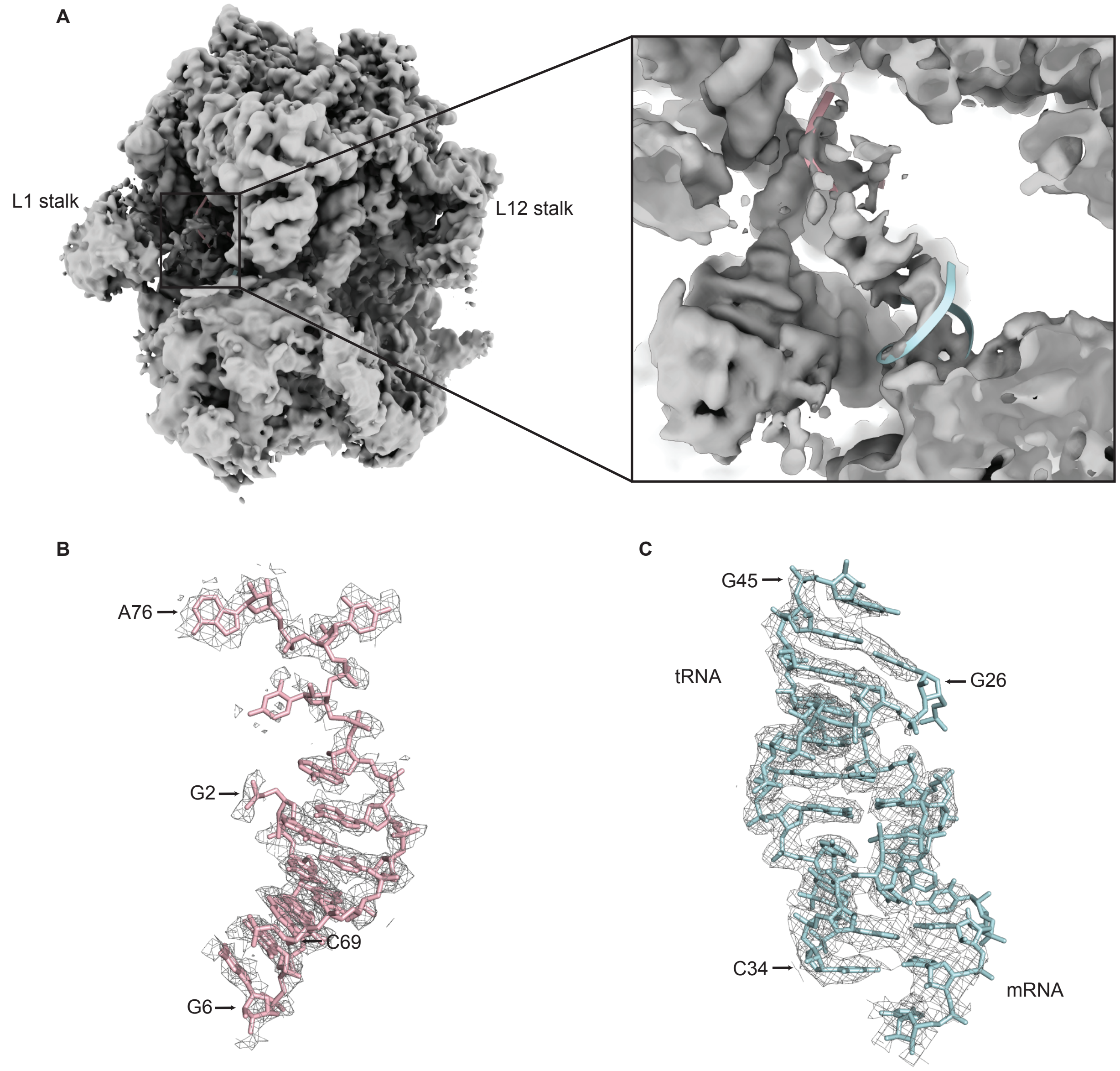

Figure S5. **CryoEM density and atomic model of E-site tRNA and mRNA, related to Figures 1-3.** A) Unsharpened consensus recon-struction of the amikacin-ribosome complex lowpass filtered to 5 Å. Weak cryoEM density is seen in the E-site of the ribosome that can fit tRNA and mRNA (ribbon). B) Atomic model of the 5' and 3' ends of E-site tRNA (light pink), derived from an E. coli fMet-tRNA starting model and fitted and refined into the sharpened and masked 50S multibody reconstruction (grey mesh). C) Atomic model of the anticodon stem-loop of the E-site tRNA, derived from an E. coli fMet-tRNA starting model, and a short polyuridine mRNA (light blue), fitted and refined into the sharpened and masked 30S head multibody reconstruction (grey mesh).

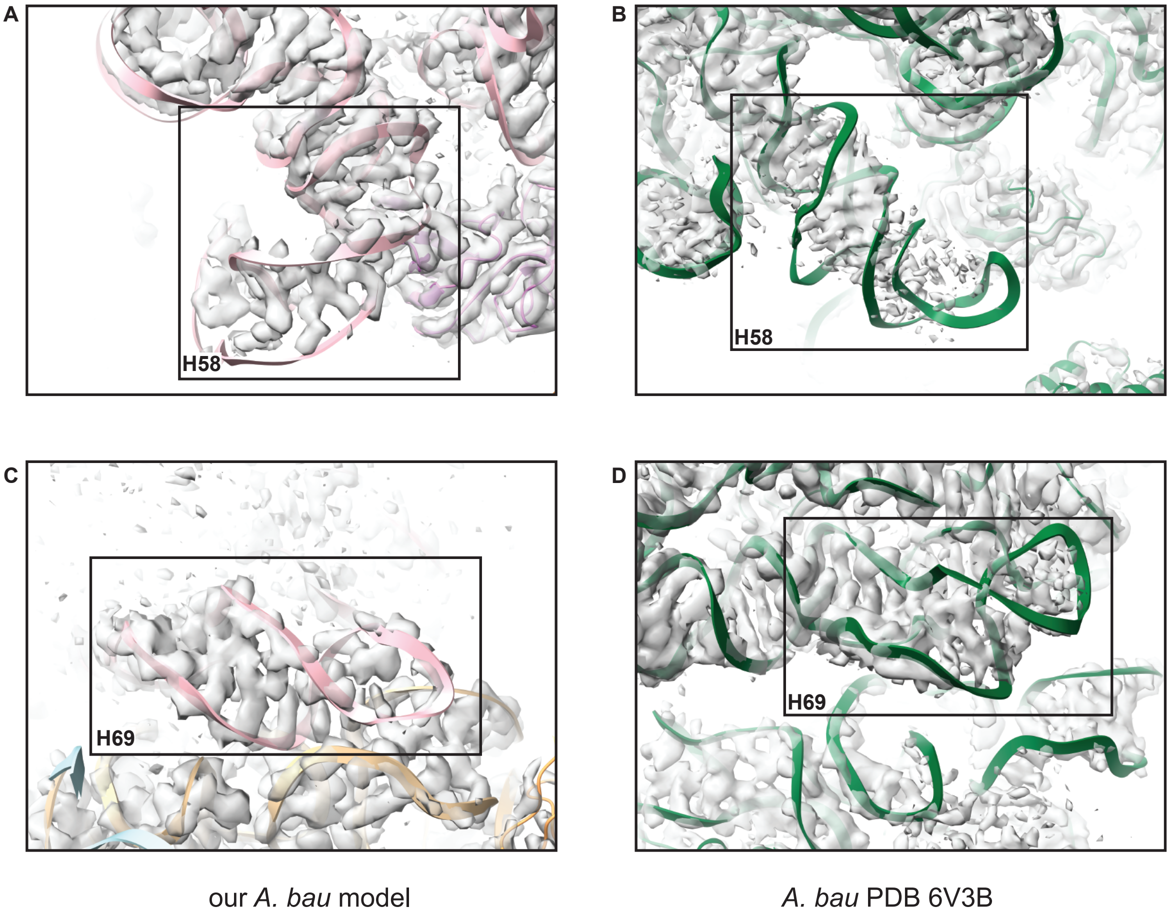

Figure S6. **EM density of 23S rRNA H58 and H69 of the *A. baumannii* ATCC 19606 and *A. baumannii* AB0057 ribosomes. Related to Figure 4.** A) H58 of the *A. baumannii* amikacin-ribosome model presented in this paper (pink, strain ATCC 19606), shown in the consensus EM map filtered by local resolution (grey). B) H58 of the alternative *A. baumannii* ribosome model (green, strain AB0057, PDB 6V3B) shown in the corresponding EM map (grey, EMD-21032). C) H69 of the *A. baumannii* amikacin-ribosome model presented in this paper (pink, strain ATCC 19606), shown in the consensus EM map filtered by local resolution (grey). D) H69 of the alternative *A. baumannii* ribosome model (green, strain AB0057, PDB 6V3B) shown in the corresponding EM map (grey, EMD-21032). In all cases, the EM density shows that the different helix conformations shown in Figure 4 correspond to the data and are not modelling errors.

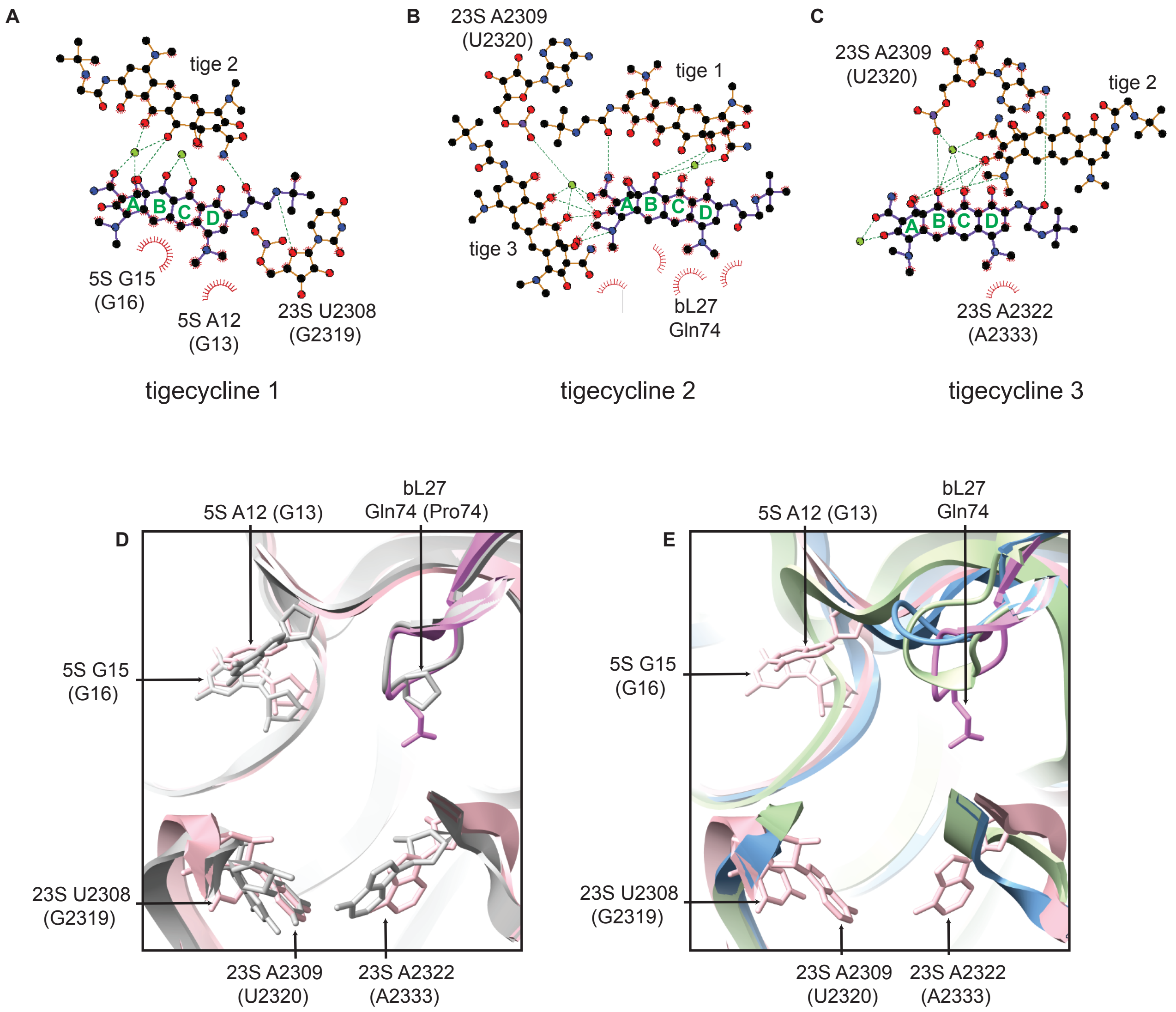

Figure S7. **The secondary tigecycline binding site. Related to Figure 7.** A-C) 2D diagrams representing the interactions that the 3 tigecycline molecules make with the surrounding ribosome. Red spikes represent hydrophobic interactions and green dashes represent polar or ionic interactions. LigPlot+ (Laskowski and Swindells, 2011) was used to generate the diagrams, which were then adapted to highlight the major interactions calculated by Arpeggio (Jubb et al., 2017). D) Atomic model of the *A. baumannii* ribosome-amikacin complex showing the secondary tigecycline binding site (with no drug bound), with residues that interact with tigecycline highlighted (pink), and atomic model of the *E. coli* ribosome with the equivalent *E. coli* residues highlighted (grey, PDB 5MDZ). Many of the residues proposed to be involved in tigecycline binding take similar conformations in the two structures. E) Atomic models of the secondary tigecycline binding site in the *A. baumannii* ribosome-amikacin complex (pink), *S. aureus* ribosome (blue, PDB 5LI0) and *T. thermophilus* ribosome (green, PDB 5E81). The rRNA surrounding the binding site and the loop in bL27, proposed to be involved in tigecycline binding, take up quite different folds in these structures. *E. coli* numbering is shown in parentheses.
